# Supplementary material for: A Systematic Investigation Unveils High Coinfection Status of Porcine Parvovirus Types 1 through 7 in China from 2016 to 2020
Source: Microbiol Spectr. 2021 Dec 1;9(3):e01294-21. doi: 10.1128/Spectrum.01294-21 (PMC8635132; doi:10.1128/Spectrum.01294-21)
Supplement: SUPPLEMENTAL FILE 1 — Supplemental material. Download SPECTRUM01294-21_Supp_1_seq12.pdf, PDF file, 0.4 MB [file spectrum01294-21_supp_1_seq12.pdf]

Table S1. Primers used for PPV1-7 detection in this study.

Virus	Primer	Sequence (5’-3’)	Location*	Size (bp)*	Gene
PPV1	PPV1-F1	CACAAGAAAAGGTTATCACCAAACAA	3571-3596	109	VP2
	PPV1-R1	GGAGTATTCAAAATTCATGTATGGTGTATT	3650-3679		
PPV2	PPV2-F1	CACGATGAGCGGTACGACGA	3173-3192	273	VP2
	PPV2-R1	ACGAGGTCTCTTCTGGGACTCCT	3423-3445		
PPV3	PPV3-F1	CAGTCTGCGCTTAACTTAGGTGATT	2555-2579	390	VP1
	PPV3-R1	TGCTTCATCCACTGGTCCCTTA	2923-2944		
PPV4	PPV4-F1	CTTTGCTTTGTCCAACGCAGA	3563-3583	488	VP2
	PPV4-R1	TAGATGTCCTGGCACAGATACTTGAC	4025-4050		
PPV5	PPV5-F1	CTTTGGTGTTGAGGGACTTAGCTTT	429-453	595	NS1
	PPV5-R1	CATGCTCCAGTGAAACATTATAACTGC	997-1023		
PPV6	PPV6-F1	AGGTTGATTCAGGGCAAATTCATA	4900-4923	689	VP2
	PPV6-R1	CCATATTGATTGATGAAGGTCTGACC	5563-5588		
PPV7	PPV7-F1	TGGTCCATGCAAGACAACGC	1308-1327	795	NS1
	PPV7-R1	GCTTGCGCAGCGTCGAT	2086-2102		

* The location and size of each amplicon were determined according to PPV1 HNLY201301 (MF447833) strain, PPV2 GX (KU745627) isolate, PPV3 GX1 (KU167028) isolate, PPV4 JS0918a (HM031134) strain, PPV5 HN01 (KF661535) isolate, PPV6 FJ2017 (MG760726) strain, and PPV7 GX49 (NC_040562) isolate, respectively.

Table S2. Distribution of PPV1-7 in 435 samples from eight provinces of China.

Province	Numbers	PPV1	PPV2	PPV3	PPV4	PPV5	PPV6	PPV7
Jiangsu	115*	+	+	+	+	+	+	+
Shandong	99	+	+	+	+	+	+	+
Fujian	71	+	+	+	+	+	+	+
Anhui	46	+	+	+	-	+	+	+
Xinjiang	32	+	+	+	-	+	+	+
Guangdong	28	+	+	+	+	+	-	-
Henan	39	+	+	-	-	+	-	+
Hebei	5	+	+	+	-	-	-	+

* The numbers indicated the numbers of samples collected from each province.

Table S3. Clinical sample infection status

Sample	Age	Region	Year	PPV1	PPV2	PPV3	PPV4	PPV5	PPV6	PPV7	PCV2
1	Suckling	Xiamen, Fujian	2016								
2	Suckling	Xiamen, Fujian	2016								+
3	Suckling	Xiamen, Fujian	2016								
7	Suckling	Xiamen, Fujian	2016								+
8	Suckling	Xiamen, Fujian	2016								+
9	Suckling	Xiamen, Fujian	2016								+
10	Suckling	Xiamen, Fujian	2016								
11	Suckling	Xiamen, Fujian	2016								+
12	Suckling	Xiamen, Fujian	2016								
13	Suckling	Xiamen, Fujian	2016								
14	Nursery	Yangzhou, Jiangsu	2016								
15	Nursery	Zhenjiang, Jiangsu	2016			+					+
16	Nursery	Yangzhou, Jiangsu	2016								+
17	Nursery	Yangzhou, Jiangsu	2016	+							+
18	Nursery	Yangzhou, Jiangsu	2016								+
20	Nursery	Yangzhou, Jiangsu	2017	+						+	+
21	Nursery	Yangzhou, Jiangsu	2017		+						
22	Nursery	Yangzhou, Jiangsu	2017		+					+	+
23	Nursery	Weifang, Shandong	2017							+	+
24	Nursery	Weifang, Shandong	2017								+
25	Nursery	Yangzhou, Jiangsu	2017		+						+
26	Nursery	Yangzhou, Jiangsu	2017								+
27	Nursery	Tianchang, Anhui	2017								+
28	Nursery	Tianchang, Anhui	2017								+
29	Suckling	Yangzhou, Jiangsu	2017	+							+
30	Nursery	Yangzhou, Jiangsu	2017	+							+
31	Nursery	Yangzhou, Jiangsu	2017								+
32	Nursery	Yangzhou, Jiangsu	2017								+
33	Nursery	Yangzhou, Jiangsu	2017		+						
46	Nursery	Weifang, Shandong	2017								+
47	Nursery	Weifang, Shandong	2017		+						+

48	Nursery	Weifang, Shandong	2017								+
49	Nursery	Weifang, Shandong	2017								+
50	Nursery	Weifang, Shandong	2017		+						+
51	Nursery	Weifang, Shandong	2017	+	+						+
52	Nursery	Weifang, Shandong	2017								+
53	Nursery	Weifang, Shandong	2017		+					+	+
54	Nursery	Weifang, Shandong	2017								+
55	Nursery	Weifang, Shandong	2017		+			+	+		+
56	Nursery	Weifang, Shandong	2017	+							+
57	Nursery	Weifang, Shandong	2017		+						+
58	Nursery	Weifang, Shandong	2017								+
59	Nursery	Weifang, Shandong	2017						+		+
60	Nursery	Weifang, Shandong	2017	+	+						+
61	Nursery	Weifang, Shandong	2017								+
62	Nursery	Weifang, Shandong	2017							+	+
63	Nursery	Weifang, Shandong	2017	+						+	+
64	Nursery	Weifang, Shandong	2017								
65	Nursery	Weifang, Shandong	2017	+							+
66	Nursery	Weifang, Shandong	2017		+			+	+		+
67	Nursery	Weifang, Shandong	2017					+			+
68	Nursery	Weifang, Shandong	2017				+		+		+
69	Nursery	Weifang, Shandong	2017	+			+			+	+
99	Nursery	Weifang, Shandong	2017								+
100	Nursery	Weifang, Shandong	2017								+
101	Nursery	Weifang, Shandong	2017								+
102	Nursery	Weifang, Shandong	2017								+
103	Nursery	Weifang, Shandong	2017	+							+
104	Nursery	Weifang, Shandong	2017			+					+
105	Nursery	Weifang, Shandong	2017								+
106	Nursery	Weifang, Shandong	2017		+					+	+
107	Nursery	Weifang, Shandong	2017		+						+
108	Nursery	Weifang, Shandong	2017		+						+
109	Nursery	Weifang, Shandong	2017		+				+		+
110	Nursery	Weifang, Shandong	2017	+							+

111	Nursery	Weifang, Shandong	2017	+	+			+		+	+
112	Nursery	Weifang, Shandong	2017		+			+			
123	Nursery	Yangzhou, Jiangsu	2018	+		+					+
124	Nursery	Yangzhou, Jiangsu	2018			+					+
125	Nursery	Yangzhou, Jiangsu	2018			+					+
126	Nursery	Yangzhou, Jiangsu	2018			+					+
127	Suckling	Yangzhou, Jiangsu	2018								+
138	Suckling	Tianchang, Anhui	2018								+
139	Nursery	Tianchang, Anhui	2018		+	+					
140	Nursery	Yancheng, Jiangsu	2018		+	+					
141	Suckling	Yancheng, Jiangsu	2018		+		+				
142	Suckling	Yangzhou, Jiangsu	2018								+
143	Suckling	Yancheng, Jiangsu	2018								+
144	Suckling	Yangzhou, Jiangsu	2018	+							+
145	Nursery	Yangzhou, Jiangsu	2018			+					+
146	Nursery	Yangzhou, Jiangsu	2018			+					+
147	Nursery	Nantong, Jiangsu	2018		+	+					
148	Nursery	Nantong, Jiangsu	2018		+	+					+
149	Nursery	Nantong, Jiangsu	2018	+	+	+					+
150	Nursery	Yangzhou, Jiangsu	2018	+		+					+
151	Nursery	Tangshan, Hebei	2018		+	+					+
152	Nursery	Tangshan, Hebei	2018		+	+				+	+
153	Nursery	Tangshan, Hebei	2018	+		+					+
154	Nursery	Tangshan, Hebei	2018	+	+	+					+
155	Nursery	Tangshan, Hebei	2018			+					+
156	Suckling	Yangzhou, Jiangsu	2018	+							+
157	Suckling	Zhenjiang, Jiangsu	2018			+					+
158	Suckling	Yangzhou, Jiangsu	2018								+
159	Suckling	Yangzhou, Jiangsu	2018			+					+
160	Suckling	Yangzhou, Jiangsu	2018								+
161	Nursery	Yangzhou, Jiangsu	2018		+	+					
162	Nursery	Yangzhou, Jiangsu	2018		+	+					
163	Suckling	Yangzhou, Jiangsu	2018								

374	Suckling	Yangzhou, Jiangsu	2018								
375	Nursery	Yangzhou, Jiangsu	2018	+							
376	Nursery	Yangzhou, Jiangsu	2018								
377	Nursery	Yangzhou, Jiangsu	2018		+					+	+
378	Nursery	Yangzhou, Jiangsu	2018		+					+	
379	Nursery	Yangzhou, Jiangsu	2018		+					+	+
428	Suckling	Yangzhou, Jiangsu	2018								
429	Suckling	Yangzhou, Jiangsu	2018								
430	Suckling	Yangzhou, Jiangsu	2018								
431	Suckling	Taizhou, Jiangsu	2018		+	+			+		+
432	Suckling	Nantong, Jiangsu	2018								
658	Nursery	Yangzhou, Jiangsu	2018	+							
659	Nursery	Yangzhou, Jiangsu	2019		+	+				+	
660	Nursery	Yangzhou, Jiangsu	2019								+
661	Nursery	Nantong, Jiangsu	2019								
662	Nursery	Yangzhou, Jiangsu	2019								
663	Nursery	Yangzhou, Jiangsu	2019								+
664	Nursery	Taizhou, Jiangsu	2019								+
665	Nursery	Yangzhou, Jiangsu	2019								+
666	Nursery	Tianchang, Anhui	2019								+
668	Nursery	Tianchang, Anhui	2019								+
708	Nursery	Yangzhou, Jiangsu	2019								
709	Nursery	Yangzhou, Jiangsu	2019								+
710	Nursery	Yangzhou, Jiangsu	2019								
711	Nursery	Yangzhou, Jiangsu	2019								
712	Nursery	Huaian, Jiangsu	2019								+
713	Nursery	Nantong, Jiangsu	2019								
717	Nursery	Yangzhou, Jiangsu	2019	+	+					+	+
718	Nursery	Yancheng, Jiangsu	2019								
720	Nursery	Taizhou, Jiangsu	2019								
733	Finishing	Liaocheng, Shandong	2020								
734	Finishing	Liaocheng, Shandong	2020								

735	Finishing	Liaocheng, Shandong	2020								
736	Finishing	Liaocheng, Shandong	2020								
737	Finishing	Liaocheng, Shandong	2020					+			
738	Finishing	Liaocheng, Shandong	2020								
739	Finishing	Liaocheng, Shandong	2020								
740	Finishing	Liaocheng, Shandong	2020					+			
741	Finishing	Liaocheng, Shandong	2020								
742	Finishing	Liaocheng, Shandong	2020							+	
743	Finishing	Liaocheng, Shandong	2020								
744	Finishing	Liaocheng, Shandong	2020								
745	Finishing	Liaocheng, Shandong	2020								+
746	Finishing	Liaocheng, Shandong	2020								
747	Finishing	Liaocheng, Shandong	2020								
748	Finishing	Liaocheng, Shandong	2020								
749	Finishing	Liaocheng, Shandong	2020								
750	Finishing	Liaocheng, Shandong	2020	+							
751	Finishing	Liaocheng, Shandong	2020							+	
752	Finishing	Liaocheng, Shandong	2020								
774	Finishing	Fuyang, Anhui	2020								
775	Finishing	Fuyang, Anhui	2020							+	+
776	Finishing	Fuyang, Anhui	2020		+			+			
777	Finishing	Fuyang, Anhui	2020		+						+
778	Finishing	Fuyang, Anhui	2020		+						
779	Finishing	Fuyang, Anhui	2020								
780	Finishing	Fuyang, Anhui	2020								
781	Finishing	Fuyang, Anhui	2020								
782	Finishing	Fuyang, Anhui	2020							+	
783	Finishing	Fuyang, Anhui	2020								+
784	Finishing	Fuyang, Anhui	2020								
785	Finishing	Fuyang, Anhui	2020								
786	Finishing	Fuyang, Anhui	2020		+	+					
787	Finishing	Fuyang, Anhui	2020								+
788	Finishing	Fuyang, Anhui	2020		+						
789	Finishing	Fuyang, Anhui	2020		+						+

790	Finishing	Fuyang, Anhui	2020	+	+			+			+
791	Finishing	Fuyang, Anhui	2020	+	+						+
792	Finishing	Fuyang, Anhui	2020	+							+
793	Finishing	Fuyang, Anhui	2020		+			+			+
794	Finishing	Fuyang, Anhui	2020								
795	Finishing	Fuyang, Anhui	2020								
796	Finishing	Fuyang, Anhui	2020								
797	Finishing	Fuyang, Anhui	2020								+
798	Finishing	Fuyang, Anhui	2020								+
799	Finishing	Fuyang, Anhui	2020		+				+		+
800	Finishing	Fuyang, Anhui	2020		+			+			+
801	Finishing	Fuyang, Anhui	2020		+	+					
802	Finishing	Fuyang, Anhui	2020		+	+			+	+	+
803	Finishing	Fuyang, Anhui	2020		+	+					
804	Finishing	Fuyang, Anhui	2020		+	+					
805	Finishing	Fuyang, Anhui	2020		+	+			+		
806	Finishing	Fuyang, Anhui	2020		+	+					+
807	Finishing	Fuyang, Anhui	2020		+						
808	Finishing	Fuyang, Anhui	2020	+	+						
809	Finishing	Fuyang, Anhui	2020								+
810	Finishing	Fuyang, Anhui	2020	+							
811	Finishing	Fuyang, Anhui	2020	+							+
812	Finishing	Fuyang, Anhui	2020		+					+	
813	Finishing	Fuyang, Anhui	2020		+					+	
814	Finishing	Qingdao, Shandong	2020								+
815	Finishing	Qingdao, Shandong	2020								+
816	Finishing	Qingdao, Shandong	2020	+							
817	Finishing	Qingdao, Shandong	2020								
818	Finishing	Qingdao, Shandong	2020	+							
819	Finishing	Qingdao, Shandong	2020								
820	Finishing	Qingdao, Shandong	2020			+					
821	Finishing	Qingdao, Shandong	2020								
822	Finishing	Qingdao, Shandong	2020								
823	Finishing	Qingdao, Shandong	2020		+						

824	Finishing	Qingdao, Shandong	2020		+						
825	Finishing	Qingdao, Shandong	2020		+						
826	Finishing	Qingdao, Shandong	2020								
827	Finishing	Qingdao, Shandong	2020		+						
828	Finishing	Qingdao, Shandong	2020								
829	Finishing	Qingdao, Shandong	2020		+	+					
830	Finishing	Qingdao, Shandong	2020	+	+						
831	Finishing	Qingdao, Shandong	2020		+						
832	Finishing	Qingdao, Shandong	2020								
833	Finishing	Qingdao, Shandong	2020							+	
834	Finishing	Nanjing, Jiangsu	2020								+
835	Finishing	Nanjing, Jiangsu	2020								
836	Finishing	Nanjing, Jiangsu	2020								
837	Finishing	Nanjing, Jiangsu	2020								
838	Finishing	Nanjing, Jiangsu	2020			+					
839	Finishing	Nanjing, Jiangsu	2020			+					
840	Finishing	Nanjing, Jiangsu	2020			+					
841	Finishing	Nanjing, Jiangsu	2020		+	+					
842	Finishing	Nanjing, Jiangsu	2020	+	+	+					
843	Finishing	Nanjing, Jiangsu	2020	+							
844	Finishing	Nanjing, Jiangsu	2020	+	+						
845	Finishing	Nanjing, Jiangsu	2020								
846	Finishing	Nanjing, Jiangsu	2020								
847	Finishing	Nanjing, Jiangsu	2020		+			+			
848	Finishing	Nanjing, Jiangsu	2020	+							
849	Finishing	Nanjing, Jiangsu	2020	+							
850	Finishing	Nanjing, Jiangsu	2020	+						+	+
851	Finishing	Nanjing, Jiangsu	2020		+			+			
852	Finishing	Nanjing, Jiangsu	2020	+						+	
853	Finishing	Nanjing, Jiangsu	2020	+						+	+
874	Finishing	Chaozhou, Guangdong	2020								
875	Finishing	Chaozhou, Guangdong	2020								
876	Finishing	Chaozhou, Guangdong	2020								
877	Finishing	Chaozhou, Guangdong	2020			+		+			

878	Finishing	Chaozhou, Guangdong	2020					+			
879	Finishing	Chaozhou, Guangdong	2020								+
880	Finishing	Chaozhou, Guangdong	2020								
881	Finishing	Chaozhou, Guangdong	2020		+			+			
882	Finishing	Chaozhou, Guangdong	2020	+							
883	Finishing	Chaozhou, Guangdong	2020								
884	Finishing	Chaozhou, Guangdong	2020								
885	Finishing	Chaozhou, Guangdong	2020	+							
886	Finishing	Chaozhou, Guangdong	2020								
887	Finishing	Chaozhou, Guangdong	2020								+
888	Finishing	Chaozhou, Guangdong	2020		+						
889	Finishing	Chaozhou, Guangdong	2020								
890	Finishing	Chaozhou, Guangdong	2020								
891	Finishing	Chaozhou, Guangdong	2020								
892	Finishing	Chaozhou, Guangdong	2020								
893	Finishing	Chaozhou, Guangdong	2020								+
894	Finishing	Chaozhou, Guangdong	2020								
895	Finishing	Chaozhou, Guangdong	2020								+
896	Finishing	Chaozhou, Guangdong	2020								
897	Finishing	Chaozhou, Guangdong	2020								
898	Finishing	Chaozhou, Guangdong	2020								
899	Finishing	Chaozhou, Guangdong	2020			+					
900	Finishing	Chaozhou, Guangdong	2020			+					+
901	Finishing	Chaozhou, Guangdong	2020			+	+				+
911	Finishing	Nanjing, Jiangsu	2020					+			+
912	Finishing	Nanjing, Jiangsu	2020								
913	Finishing	Nanjing, Jiangsu	2020								
914	Finishing	Nanjing, Jiangsu	2020								
915	Finishing	Nanjing, Jiangsu	2020								
916	Finishing	Nanjing, Jiangsu	2020								
917	Finishing	Nanjing, Jiangsu	2020			+					+
922	Finishing	Fuzhou, Fujian	2020			+					
923	Finishing	Fuzhou, Fujian	2020			+					+
924	Finishing	Fuzhou, Fujian	2020			+			+	+	

925	Finishing	Fuzhou, Fujian	2020			+					
926	Finishing	Fuzhou, Fujian	2020			+					
927	Finishing	Fuzhou, Fujian	2020								+
928	Finishing	Fuzhou, Fujian	2020		+	+				+	
929	Finishing	Fuzhou, Fujian	2020			+					
930	Finishing	Fuzhou, Fujian	2020			+				+	
931	Finishing	Fuzhou, Fujian	2020			+					
932	Finishing	Fuzhou, Fujian	2020			+					
933	Finishing	Fuzhou, Fujian	2020			+					
934	Finishing	Fuzhou, Fujian	2020			+				+	
935	Finishing	Fuzhou, Fujian	2020			+					+
936	Finishing	Fuzhou, Fujian	2020			+					
937	Finishing	Fuzhou, Fujian	2020							+	
938	Finishing	Fuzhou, Fujian	2020								+
939	Finishing	Fuzhou, Fujian	2020					+			
940	Finishing	Fuzhou, Fujian	2020		+						
941	Finishing	Fuzhou, Fujian	2020		+	+		+			
942	Finishing	Fuzhou, Fujian	2020		+	+					
943	Finishing	Fuzhou, Fujian	2020					+	+		
944	Finishing	Fuzhou, Fujian	2020								
945	Finishing	Fuzhou, Fujian	2020		+	+	+			+	+
946	Finishing	Fuzhou, Fujian	2020		+	+	+				
947	Finishing	Fuzhou, Fujian	2020		+	+		+			
948	Finishing	Fuzhou, Fujian	2020		+	+					
949	Finishing	Fuzhou, Fujian	2020		+						
950	Finishing	Fuzhou, Fujian	2020		+	+	+			+	+
951	Finishing	Fuzhou, Fujian	2020			+					
952	Finishing	Fuzhou, Fujian	2020		+	+					
953	Finishing	Fuzhou, Fujian	2020		+		+				+
954	Finishing	Fuzhou, Fujian	2020					+		+	
955	Finishing	Fuzhou, Fujian	2020								
956	Finishing	Fuzhou, Fujian	2020								+
957	Finishing	Fuzhou, Fujian	2020	+			+				
958	Finishing	Fuzhou, Fujian	2020	+							

959	Finishing	Fuzhou, Fujian	2020	+	+		+				
960	Finishing	Fuzhou, Fujian	2020	+			+			+	+
961	Finishing	Fuzhou, Fujian	2020	+		+		+	+	+	+
962	Finishing	Fuzhou, Fujian	2020			+	+				
963	Finishing	Fuzhou, Fujian	2020								
964	Finishing	Fuzhou, Fujian	2020			+				+	
965	Finishing	Fuzhou, Fujian	2020	+		+	+				
966	Finishing	Fuzhou, Fujian	2020			+	+				
967	Finishing	Fuzhou, Fujian	2020	+			+				
968	Finishing	Fuzhou, Fujian	2020	+							+
969	Finishing	Fuzhou, Fujian	2020								
970	Finishing	Fuzhou, Fujian	2020			+					
971	Finishing	Fuzhou, Fujian	2020			+				+	
972	Finishing	Fuzhou, Fujian	2020			+					
973	Finishing	Fuzhou, Fujian	2020								
974	Finishing	Fuzhou, Fujian	2020								
975	Finishing	Fuzhou, Fujian	2020				+	+			
976	Finishing	Fuzhou, Fujian	2020							+	
977	Finishing	Fuzhou, Fujian	2020				+			+	
978	Finishing	Fuzhou, Fujian	2020								
979	Finishing	Fuzhou, Fujian	2020			+	+	+	+		
980	Finishing	Fuzhou, Fujian	2020			+		+			
981	Finishing	Fuzhou, Fujian	2020								
982	Finishing	Fuzhou, Fujian	2020				+				
1028	Finishing	Zibo, Shandong	2020								
1029	Finishing	Zibo, Shandong	2020							+	
1030	Finishing	Zibo, Shandong	2020								
1031	Finishing	Zibo, Shandong	2020								
1032	Finishing	Zibo, Shandong	2020								
1033	Finishing	Zibo, Shandong	2020								
1034	Finishing	Zibo, Shandong	2020								
1035	Finishing	Zibo, Shandong	2020								
1036	Finishing	Zibo, Shandong	2020								
1037	Finishing	Zibo, Shandong	2020								

1038	Finishing	Zibo, Shandong	2020								
1039	Finishing	Zibo, Shandong	2020								
1040	Finishing	Zibo, Shandong	2020								
1041	Finishing	Zibo, Shandong	2020								
1042	Finishing	Zibo, Shandong	2020	+							
1043	Finishing	Zibo, Shandong	2020	+							
1044	Finishing	Zibo, Shandong	2020	+							
1045	Finishing	Zibo, Shandong	2020	+							
1046	Finishing	Zibo, Shandong	2020	+							
1066	Finishing	Zhumadian, Henan	2020					+			+
1067	Finishing	Zhumadian, Henan	2020	+							
1068	Finishing	Zhumadian, Henan	2020								
1069	Finishing	Zhumadian, Henan	2020								
1070	Finishing	Zhumadian, Henan	2020								
1071	Finishing	Zhumadian, Henan	2020								
1072	Finishing	Zhumadian, Henan	2020								+
1073	Finishing	Zhumadian, Henan	2020								+
1074	Finishing	Zhumadian, Henan	2020								
1075	Finishing	Zhumadian, Henan	2020							+	+
1076	Finishing	Zhumadian, Henan	2020					+			
1077	Finishing	Zhumadian, Henan	2020								
1078	Finishing	Zhumadian, Henan	2020								
1079	Finishing	Zhumadian, Henan	2020								
1080	Finishing	Zhumadian, Henan	2020								
1081	Finishing	Zhumadian, Henan	2020								+
1082	Finishing	Zhumadian, Henan	2020					+		+	
1083	Finishing	Zhumadian, Henan	2020					+		+	+
1084	Finishing	Zhumadian, Henan	2020								+
1085	Finishing	Zhumadian, Henan	2020					+			+
1086	Finishing	Zhumadian, Henan	2020							+	
1087	Finishing	Zhumadian, Henan	2020					+			+
1088	Finishing	Zhumadian, Henan	2020		+						
1089	Finishing	Zhumadian, Henan	2020								
1090	Finishing	Zhumadian, Henan	2020								+

1091	Finishing	Zhumadian, Henan	2020	+					+	
1092	Finishing	Zhumadian, Henan	2020							
1093	Finishing	Zhumadian, Henan	2020							
1094	Finishing	Zhumadian, Henan	2020							
1095	Finishing	Zhumadian, Henan	2020							+
1096	Finishing	Zhumadian, Henan	2020							
1097	Finishing	Zhumadian, Henan	2020				+			
1098	Finishing	Zhumadian, Henan	2020							+
1099	Finishing	Zhumadian, Henan	2020				+			
1100	Finishing	Zhumadian, Henan	2020							+
1101	Finishing	Zhumadian, Henan	2020				+			
1102	Finishing	Zhumadian, Henan	2020				+			
1103	Finishing	Zhumadian, Henan	2020							
1104	Finishing	Zhumadian, Henan	2020				+			
1125	Finishing	Taizhou, Jiangsu	2020							
1126	Finishing	Taizhou, Jiangsu	2020							
1127	Finishing	Taizhou, Jiangsu	2020							
1128	Finishing	Taizhou, Jiangsu	2020							+
1129	Finishing	Taizhou, Jiangsu	2020							+
1130	Finishing	Taizhou, Jiangsu	2020		+				+	
1131	Finishing	Taizhou, Jiangsu	2020							+
1132	Finishing	Taizhou, Jiangsu	2020	+					+	
1133	Finishing	Taizhou, Jiangsu	2020							
1134	Finishing	Taizhou, Jiangsu	2020	+					+	+
1135	Finishing	Taizhou, Jiangsu	2020		+				+	
1136	Finishing	Taizhou, Jiangsu	2020	+	+					
1137	Finishing	Taizhou, Jiangsu	2020	+					+	
1138	Finishing	Taizhou, Jiangsu	2020	+						+
1139	Finishing	Taizhou, Jiangsu	2020							+
1140	Finishing	Taizhou, Jiangsu	2020						+	+
1141	Finishing	Taizhou, Jiangsu	2020						+	
1142	Finishing	Taizhou, Jiangsu	2020	+					+	
1143	Finishing	Taizhou, Jiangsu	2020							+
1144	Finishing	Taizhou, Jiangsu	2020						+	+

1200	Nursery	Kashi, Xinjiang	2020								
1201	Nursery	Kashi, Xinjiang	2020								
1202	Nursery	Kashi, Xinjiang	2020								
1203	Nursery	Kashi, Xinjiang	2020								
1204	Nursery	Kashi, Xinjiang	2020							+	
1205	Nursery	Kashi, Xinjiang	2020							+	
1206	Nursery	Kashi, Xinjiang	2020								
1207	Nursery	Kashi, Xinjiang	2020								
1208	Nursery	Kashi, Xinjiang	2020								
1209	Nursery	Kashi, Xinjiang	2020								
1210	Nursery	Kashi, Xinjiang	2020							+	
1211	Nursery	Kashi, Xinjiang	2020			+				+	
1212	Nursery	Kashi, Xinjiang	2020	+						+	+
1213	Nursery	Kashi, Xinjiang	2020			+		+			+
1214	Nursery	Kashi, Xinjiang	2020		+	+		+			+
1215	Nursery	Kashi, Xinjiang	2020			+		+		+	
1216	Nursery	Kashi, Xinjiang	2020		+	+			+	+	
1217	Nursery	Kashi, Xinjiang	2020							+	
1218	Nursery	Kashi, Xinjiang	2020	+		+					
1219	Nursery	Kashi, Xinjiang	2020	+		+					
1220	Nursery	Kashi, Xinjiang	2020	+							
1221	Nursery	Kashi, Xinjiang	2020	+		+				+	
1222	Nursery	Kashi, Xinjiang	2020			+					
1223	Nursery	Kashi, Xinjiang	2020			+					
1224	Nursery	Kashi, Xinjiang	2020	+	+	+				+	
1225	Nursery	Kashi, Xinjiang	2020	+	+	+					
1226	Nursery	Kashi, Xinjiang	2020	+	+	+					+
1227	Nursery	Kashi, Xinjiang	2020	+	+	+			+	+	+
1228	Nursery	Kashi, Xinjiang	2020	+	+	+			+	+	+
1229	Nursery	Kashi, Xinjiang	2020	+		+			+		+
1230	Suckling	Hutubi, Xinjiang	2020	+		+					+
1231	Nursery	Changji, Xinjiang	2020	+		+					+
1232	Nursery	Yancheng, Jiangsu	2020								
435				70	98	98	19	40	17	67	166

Table S4. Cross-over event in PPV7 HBTZ20180519-152 strain identified by RDP4.

Recombined virus	Parental virus		Breakpoints*		Score for the seven detection methods embedded in RDP4[#]						
	Major	Minor	Begin	End	RDP	GENECONV	BootScan	MaxChi	Chimaera	SiScan	3Seq
HBTZ20180519-152	JX15	JX38	600	1648	9.4×10^{-19}	3.2×10^{-22}	5.4×10^{-23}	6.7×10^{-19}	6.3×10^{-6}	7.0×10^{-20}	1.2×10^{-4}

* The breakpoints are based on the locations in the genome of PPV7 HBTZ20180519-152 strain.

# The *p* value cut-off is set at 0.05. $p<0.05$ indicates the cross-over events are significant.

Supplementary figure legends

Figure S1. Establishment of a new panel of PPV1 through PPV7 PCR assays. Corresponding amplicons of PPV1 through PPV7 could be specifically produced. M, DNA ladder; Neg, negative control.

Figure S2. The specificity of the panel of PPV1-7 PCR assays. Specific amplicons could be produced only when the corresponding PPV1 through PPV7 strains were detected. No amplicons could be generated when other pathogens were tested. The representative specificity results for each assay were shown. Lane 1, PPV1; lane 2, PPV2; lane 3, PPV3; lane 4, PPV4; lane 5, PPV5; lane 6, PPV6; lane 7, PPV7; lane 8, PCV1 JSNJ2004-913; lane 9, PCV2 JSYZ1705-32; lane 10, PCV3 SD17-36; lane 11, PCV4 JSYZ1901-2; lane 12, PRV XJ03; lane 13, PEDV XM2-4; lane 14, CSFV JS1805-2; lane 15, PRRSV1 HLJB1; lane 16, PRRSV2 SD17-38; lane 17, Negative control.

Figure S3. The sensitivity of the panel of PPV1-7 PCR assays. The positive control plasmid was diluted from 1.8×10^7 copies/μl to 1.8×10^1 copies/μl. The dilutions were used to determine the detection limits of each genotype of PPV PCR methods. The detection limits for PPV1-7 PCR assays are between 1.8×10^1 copies/μl and 1.8×10^3 copies/μl.

Figure S4. The reproducibility of the panel of PPV1-7 PCR assays. The panel of PPV1-7 PCR assays was used to detection two concentrations (1.8×10^5 copies/μl and 1.8×10^3 copies/μl) of the positive plasmid by two individuals. Specific amplicons could be generated using different concentrations by different operators.

Figure S5. Genome homology and variation distribution. Based on the comparison of our nearly complete PPV genomes and representative genomes of each genotype, the genome homologies of PPV1 through PPV7 have been presented.

Figure S6. Sequence comparison of PPV2 VP2 proteins. The overall VP2 similarity and polymorphic sites were shown.

Figure S7. Sequence comparison of PPV3 VP2 proteins. The overall VP2 similarity and polymorphic sites were shown.

Figure S8. Sequence comparison of PPV4 VP2 proteins. The overall VP2 similarity and polymorphic sites were shown.

Figure S9. Sequence comparison of PPV5 VP2 proteins. The overall VP2 similarity and polymorphic sites were shown.

Figure S10. Sequence comparison of PPV6 VP2 proteins. The overall VP2 similarity and polymorphic sites were shown.

Figure S11. Sequence comparison of PPV7 VP2 proteins. The overall VP2 similarity and polymorphic sites were shown.

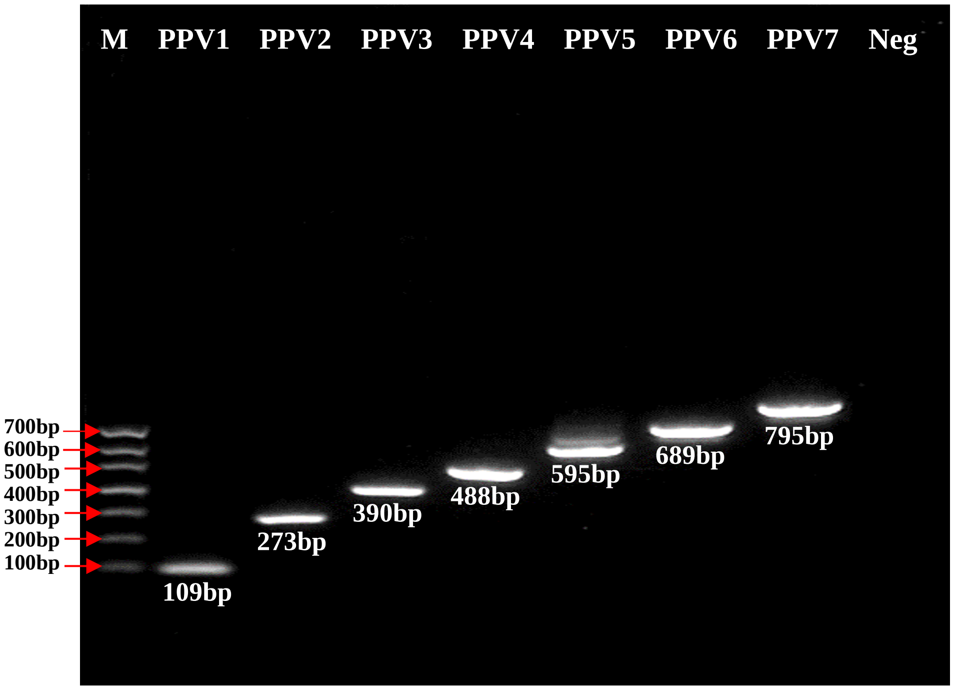
M
PPV1
PPV2
PPV3
PPV4
PPV5
PPV6
PPV7
Neg
700bp
600bp
500bp
400bp
300bp
200bp
100bp
109bp
273bp
390bp
488bp
595bp
689bp
795bp

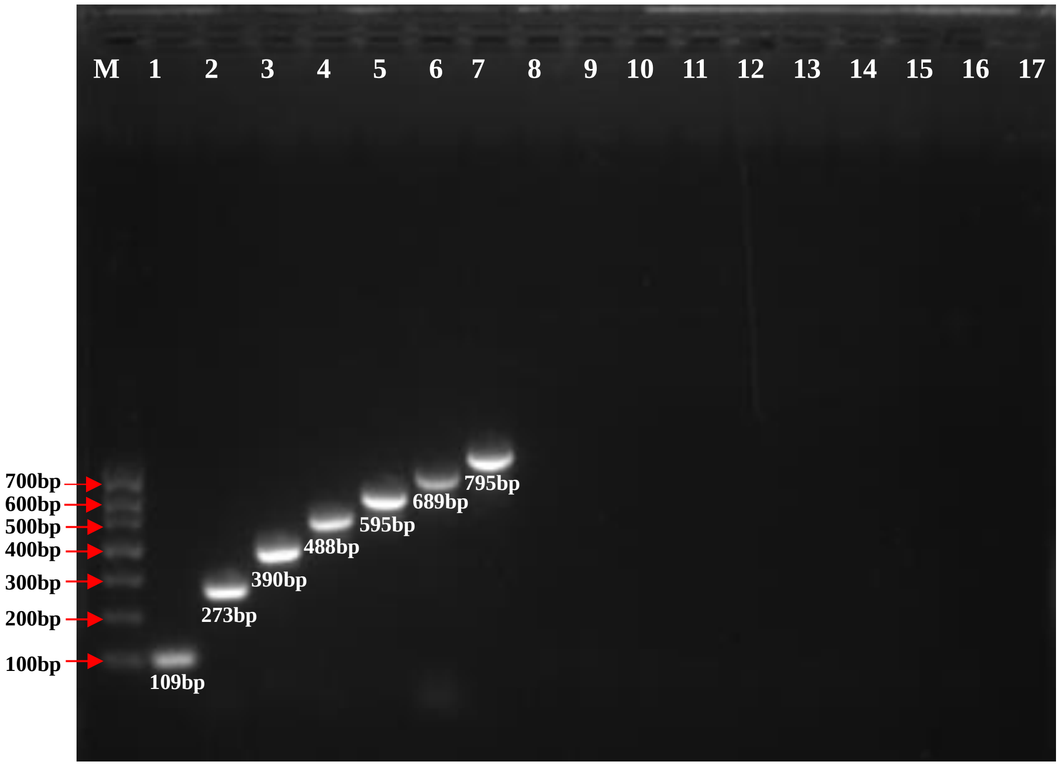
M
1
2
3
4
5
6
7
8
9
10
11
12
13
14
15
16
17
700bp
600bp
500bp
400bp
300bp
200bp
100bp
109bp
273bp
390bp
488bp
595bp
689bp
795bp

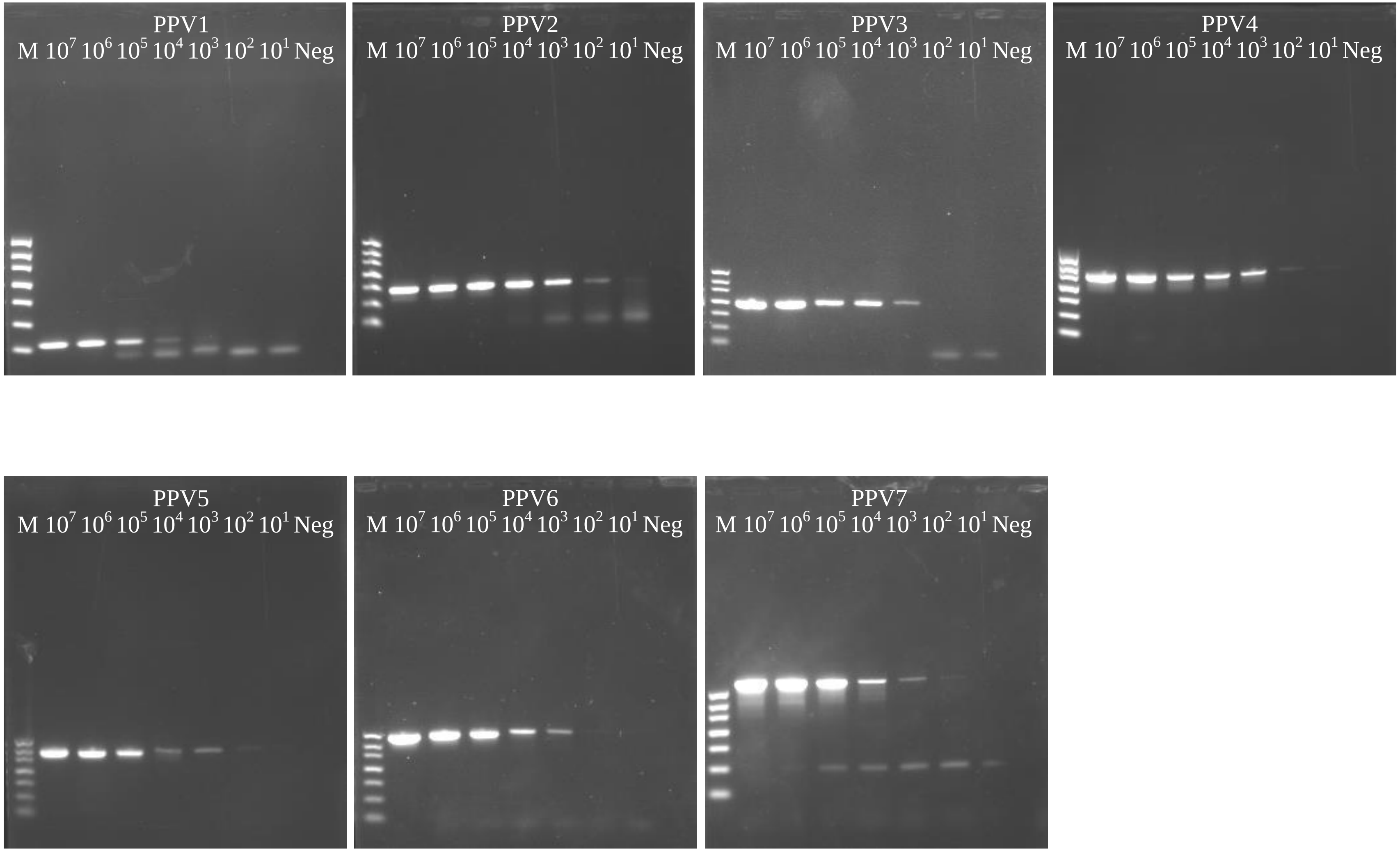
PPV1
M 10^7 10^6 10^5 10^4 10^3 10^2 10^1 Neg
PPV2
M 10^7 10^6 10^5 10^4 10^3 10^2 10^1 Neg
PPV3
M 10^7 10^6 10^5 10^4 10^3 10^2 10^1 Neg
PPV4
M 10^7 10^6 10^5 10^4 10^3 10^2 10^1 Neg
PPV5
M 10^7 10^6 10^5 10^4 10^3 10^2 10^1 Neg
PPV6
M 10^7 10^6 10^5 10^4 10^3 10^2 10^1 Neg
PPV7
M 10^7 10^6 10^5 10^4 10^3 10^2 10^1 Neg

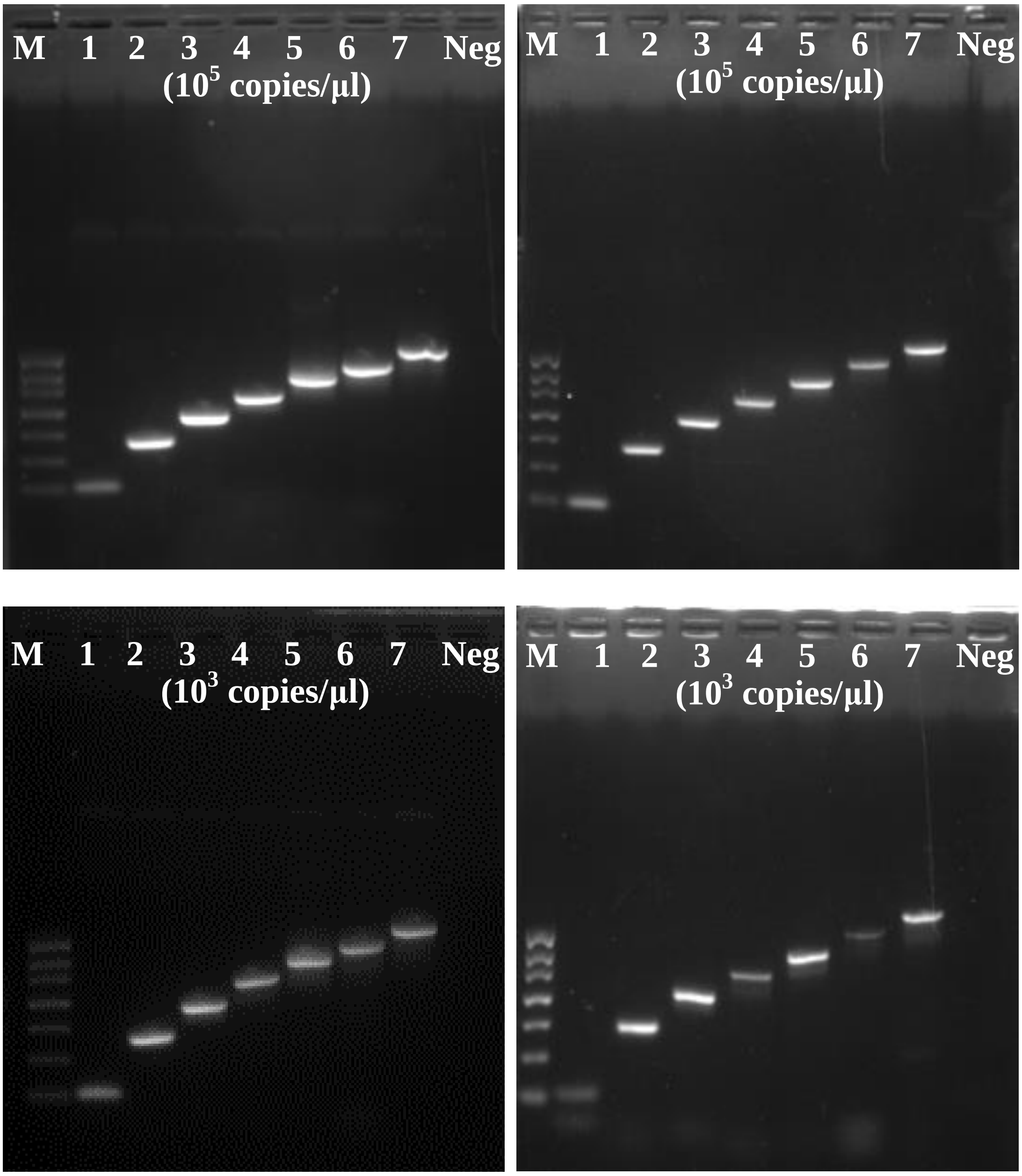
M 1 2 3 4 5 6 7 Neg
(10^5 copies/µl)
M 1 2 3 4 5 6 7 Neg
(10^5 copies/µl)
M 1 2 3 4 5 6 7 Neg
(10^3 copies/µl)
M 1 2 3 4 5 6 7 Neg
(10^3 copies/µl)

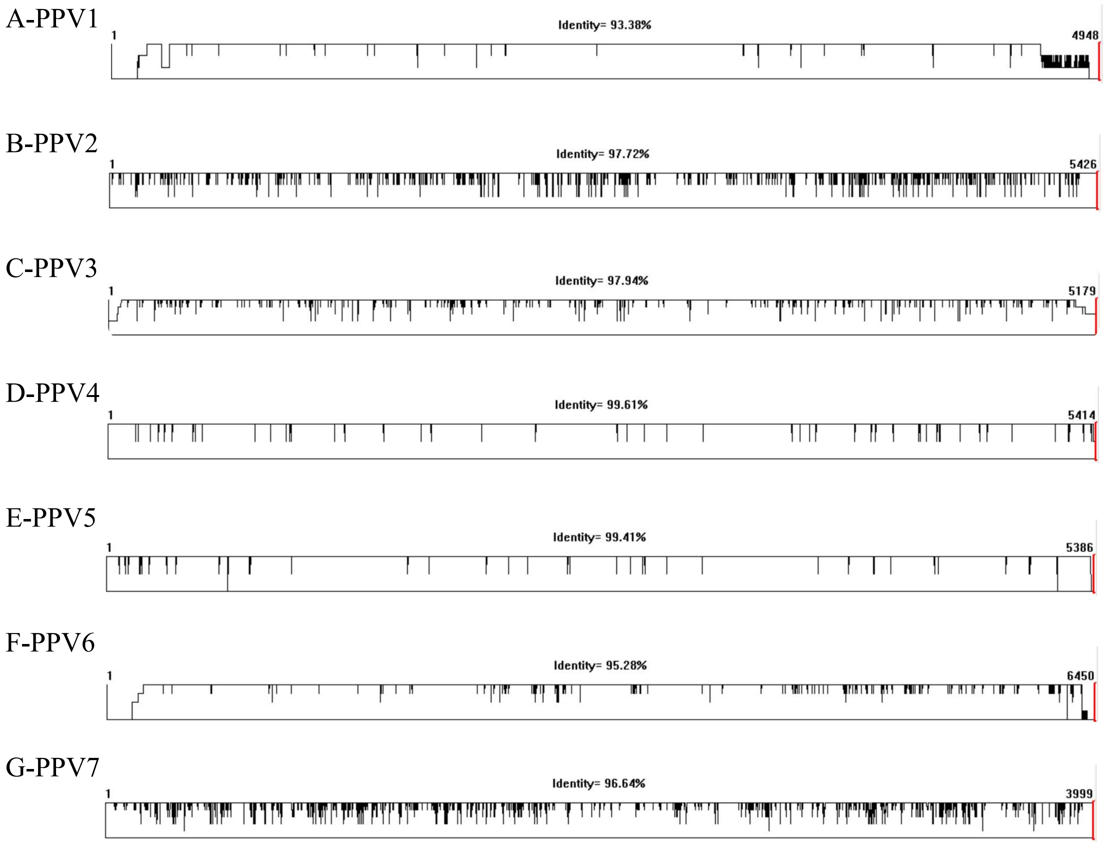
A-PPV1
Identity= 93.38%
1
4948
B-PPV2
Identity= 97.72%
1
5426
C-PPV3
Identity= 97.94%
1
5179
D-PPV4
Identity= 99.61%
1
5414
E-PPV5
Identity= 99.41%
1
5386
F-PPV6
Identity= 95.28%
1
6450
G-PPV7
Identity= 96.64%
1
3999

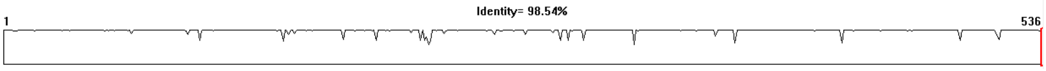


```
PPV2-NC025965 MVILSPKTDYKPLFLDADTSKFDSEPGMGFLTPWQYFDFNCYMNHFTPSDWQELGRRYDSIRPKSLTISVENVVIKDVHQTNNETNVHDSGTGGIMIFED 100
PPV2-MH921914 ...............................................................................................L.... 100
PPV2-KU745627 ..................................................................S............................L.... 100
PPV2-MK092395 .................................................................................................... 100
PPV2-MK092390 .................................................................................................... 100
PPV2-MK092397 .................................................................................................... 100
PPV2-MK092399 ................................................................P................................... 100
PPV2-MK092393 .................................................................................................... 100
PPV2-MK092396 .................................................................................................... 100
PPV2-MK092389 .................................................................................................... 100
PPV2-MK092391 .................................................................................................... 100
PPV2-MK092398 .................................................................................................... 100
PPV2-MK092394 .........................................................C....................................V..... 100
PPV2-MK092392 .......A..............................................E............................................. 100
PPV2-KM926355 .................................................................................................... 100
PPV2-JX101462 .....S.............................................................................................. 100
PPV2-MK092386 ..............................V.....................K............................................... 100
PPV2-JX101461 .................................................................................................... 100
PPV2-KY018935 .................................................................................................... 100
PPV2-KY018936 .................................................................................................... 100
PPV2-MK092388 ..................................L................................................................. 100
PPV2-MK092401 ................S................................................................................... 100
PPV2-MK092402 .................................................................................................... 100
PPV2-MK092385 .................................................................................................... 100
PPV2-MK092400 ......R............................................................................................. 100
PPV2-MK092387 .................................................................................................... 100
PPV2-KP765690 .................................................................................................... 100
PPV2-KY586144 .................................................................................................... 100
PPV2-112      ..................................................................S............................L.... 100
PPV2-717      .................................................................................................... 100
PPV2-1137     ..................................................................S............................L.... 100

PPV2-NC025965 SEYTFPYVIGHAQEGNPGALSIQWYNPPQYAYFTGFNPIAWDHANGTTKYRVHPSADTEFFVLEEHAAQILRSGDGTSFAYEFPSLEPKRLGSRMGTLNL 200
PPV2-MH921914 ...............................................I..Q.......................................I.....V... 200
PPV2-KU745627 ...............................................I.................................................... 200
PPV2-MK092395 .................................................................................................... 200
PPV2-MK092390 ...................................................................................................P 200
PPV2-MK092397 .................................................................................................... 200
PPV2-MK092399 .................................................................................................... 200
PPV2-MK092393 .................................................................................................... 200
PPV2-MK092396 ..C.....T........................................................................................... 200
PPV2-MK092389 .................................................................................................... 200
PPV2-MK092391 .................................................................................................... 200
PPV2-MK092398 .................................................................................................... 200
PPV2-MK092394 ............................................................................................R....... 200
PPV2-MK092392 .................................................................................................... 200
PPV2-KM926355 .................................................................................................... 200
PPV2-JX101462 .........................................G..............N.............................D............. 200
PPV2-MK092386 ............................................T..............C........................................ 200
PPV2-JX101461 .................................................................................Q.................. 200
PPV2-KY018935 .................................................................................................... 200
PPV2-KY018936 .................................................................................................... 200
PPV2-MK092388 .D..........................................T..............................A................T....... 200
PPV2-MK092401 .D..........................................T..............................A................T....... 200
PPV2-MK092402 .D..........................................T..............................A................T....... 200
PPV2-MK092385 .D..........................................T..............................A................T....... 200
PPV2-MK092400 .D..........................................T..............................A................T....... 200
PPV2-MK092387 .D..........................................T..............................A................T....... 200
PPV2-KP765690 .D..........................................S..............................A................T....... 200
PPV2-KY586144 .D..........................................S....................................................... 200
PPV2-112      ..............................................SI..Q................................................. 200
PPV2-717      .D..........................................T..............................A................T....... 200
PPV2-1137     ...............................................I..Q................................................. 200

PPV2-NC025965 RHNPVLPSRLAIYLGQDGSDAPTFYQPQGTDLDIFPQGFIPGPRPCLPVSTQLRASSDFDEMSAIAYGDPTSNNRHSLMPFTRQATTISTQNYNRQGEVE 300
PPV2-MH921914 ..........T.......NN.......R.........................S...............R.............................. 300
PPV2-KU745627 ..................NN.................................S...............R.............................. 300
PPV2-MK092395 .......................S............................................................................ 300
PPV2-MK092397 .............................................................................................S...... 300
PPV2-MK092399 ..................................S..............P.............T...................R................ 300
PPV2-MK092393 ........................................................I..........................R................ 300
PPV2-MK092396 .................................................................................................... 300
PPV2-MK092389 .................................................................................................... 300
PPV2-MK092391 .................................................................................................... 300
PPV2-MK092398 .................................................................................................... 300
PPV2-MK092394 ..........V......................................................................................... 300
PPV2-MK092392 ..................F......................................G.......................................... 300
PPV2-KM926355 .................................................................................................... 300
PPV2-JX101462 .................................................................................................... 300
PPV2-MK092386 ............................R.....................................................................I. 300
PPV2-JX101461 .................................................................................................... 300
PPV2-KY018935 .................................................................................................... 300
PPV2-KY018936 .................................................................................................... 300
PPV2-MK092388 ...............E.S.NG..................................................................M...S.......Q 300
PPV2-MK092401 ...............E.S.NG..................................................................M...S.......Q 300
PPV2-MK092402 ...............E.S.NG..................................................................M...S.......Q 300
PPV2-MK092385 ...............E.S.NG..................................................................M...S.......Q 300
PPV2-MK092400 ...............E.S.NG...............................P..................................M...S.......Q 300
PPV2-MK092387 ...............E.S.NG..................................................................M...S.......Q 300
PPV2-KP765690 ...............E.Q.NG..................................................................M...S.......Q 300
PPV2-KY586144 ...............E.QTNG..................................................................M...S.......Q 300
```

```
PPV2-112      ..................NNS......R.........................S...............R.............................. 300
PPV2-717      ...............E.S.NG..................................................................M...S.......Q 300
PPV2-1137     ..................NN.......R.........................S...............R.............................. 300

PPV2-NC025965 RNVHFQLGDMAFARSSAEDSFYERFEEDKDYRNPGGYVKKPRPLVTAEREGLGERPGDALMVPTWGAHLPGSSTGPGTTKTEKVSLPFIPPMPGACWDER 400
PPV2-MH921914 .........................D.........................................K.........P...................... 400
PPV2-KU745627 .........................D.........................................K.........A...................... 400
PPV2-MK092395 .................................................................................................... 400
PPV2-MK092390 .................................................................................................... 400
PPV2-MK092397 .................................................................................................... 400
PPV2-MK092399 .................................................................................................... 400
PPV2-MK092393 .................................................................................................... 400
PPV2-MK092396 .................................................................................................... 400
PPV2-MK092389 .................................................................................................... 400
PPV2-MK092391 .................................................................................................... 400
PPV2-MK092398 .................................................................................................... 400
PPV2-MK092394 .................................................................................................... 400
PPV2-MK092392 .................................................................................................... 400
PPV2-KM926355 .................................................................................................... 400
PPV2-JX101462 .................................................................................................... 400
PPV2-MK092386 .................................................K.................................................. 400
PPV2-JX101461 .................................................................................................... 400
PPV2-KY018935 .................................................................................................... 400
PPV2-KY018936 ...................................................................Q................................ 400
PPV2-MK092388 .........................D...................................................I...................... 400
PPV2-MK092401 .........................D...................................................I...................... 400
PPV2-MK092402 .........................D...............................G...................I...................... 400
PPV2-MK092385 .........................D...................................................I...................... 400
PPV2-MK092400 .........................D...................................................I...................... 400
PPV2-MK092387 .........................D...................................................I...................... 400
PPV2-KP765690 .........................D.......................................................................... 400
PPV2-KY586144 .........................D.E........................................................................ 400
PPV2-112      .........................D.........................................K.........A...................... 400
PPV2-717      .........................D...................................................I...................... 400
PPV2-1137     .........................D.........................................K.........P...................... 400

PPV2-NC025965 PLCYEDDIWCKKPYTDCSFMSEKNNLGAWALVDPPPQVFFRMQPQVGPPPADLDQRTFLPPALNQYAMFTVSYTMEWVCEPRKHTRRHNLEPPPPMPYTE 500
PPV2-MH921914 .................................................................................................... 500
PPV2-KU745627 .................................................................................................... 500
PPV2-MK092395 ................................N................................................................... 500
PPV2-MK092390 ................................N................................................................... 500
PPV2-MK092397 ................................N................................................................... 500
PPV2-MK092399 ................................N................................................................... 500
PPV2-MK092393 ..................S.............N................................................................... 500
PPV2-MK092396 ................................N................................................................... 500
PPV2-MK092389 ................................N................................................................... 500
PPV2-MK092391 ................................N................................................................... 500
PPV2-MK092398 ................................N................................................................... 500
PPV2-MK092394 ................................N.....S............................................................. 500
PPV2-MK092392 ................................N...................................L............................... 500
PPV2-KM926355 .................................................................................................... 500
PPV2-JX101462 .................................................................................................... 500
PPV2-MK092386 .................................................................................................... 500
PPV2-JX101461 .................................................................................................... 500
PPV2-KY018935 .................................................................................................... 500
PPV2-KY018936 .................................................................................................... 500
PPV2-MK092388 .............................................................................................A...... 500
PPV2-MK092401 .............................................................................................A...... 500
PPV2-MK092402 ....................................................F........................................A...... 500
PPV2-MK092385 .............................................................................................A...... 500
PPV2-MK092400 .............................................................................................A...... 500
PPV2-MK092387 .............................................................................................A...... 500
PPV2-KP765690 .............................................................................................A...... 500
PPV2-KY586144 .............................................................................................A...... 500
PPV2-112      .................................................................................................... 500
PPV2-717      ..................................................................S..........................A...... 500
PPV2-1137     .................................................................................................... 500

PPV2-NC025965 SGDPPFLLTRSHASNDYPRYSLPVEAFRPEGRAHRV 536
PPV2-MH921914 ............VT...................... 536
PPV2-KU745627 ............LT....................C. 536
PPV2-MK092395 .................................... 536
PPV2-MK092390 .................................... 536
PPV2-MK092397 .................................... 536
PPV2-MK092399 .................................... 536
PPV2-MK092393 .................................... 536
PPV2-MK092396 .................................... 536
PPV2-MK092389 .................................... 536
PPV2-MK092391 .................................... 536
PPV2-MK092398 .................................... 536
PPV2-MK092394 .................................... 536
PPV2-MK092392 .................................... 536
PPV2-KM926355 .................................... 536
PPV2-JX101462 .................................... 536
PPV2-MK092386 .................................... 536
PPV2-JX101461 .............T...................... 536
PPV2-KY018935 .............T...................... 536
PPV2-KY018936 .............T...................... 536
PPV2-MK092388 .................................... 536
PPV2-MK092401 ...........N........................ 536
PPV2-MK092402 .................................... 536
PPV2-MK092385 .................................... 536
PPV2-MK092400 .................................... 536
PPV2-MK092387 .................................... 536
PPV2-KP765690 .............T...................... 536
PPV2-KY586144 .................................... 536
PPV2-112      ............VT...................... 536
PPV2-717      .................................... 536
PPV2-1137     ............VT...................... 536
```

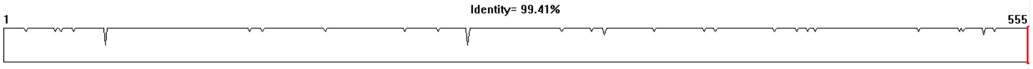


```
PPV3-MK092412 MAAPVTGATGGGIKVKAQWLGGTHFSDNTIVTSHTRTSMLADRGGYVPVYRSGSHVSDRQPVMGMRTPYSYIDVNAISAHLTPRDFQQLLDEYEEIRPKK 100
PPV3-MK092410 .................................................................................................... 100
PPV3-KU167028 .......................................................T............................................ 100
PPV3-KU167029 .......................................................T............................................ 100
PPV3-KY586145 ............V....................................................................................... 100
PPV3-MK092409 .................................................................................................... 100
PPV3-MK092411 ...............................A......T............................................................. 100
PPV3-151      .................................................................................................... 100
PPV3-155      .......................................................T............................................ 100
PPV3-829      .......................................................T............................................ 100
PPV3-838      ............................R..........................T............................................ 100

PPV3-MK092412 LVIGISGIVIKDVSVTTTGTTVSDSASGGITVFSDDAYDYPYVLGHNQDTLPGHLPGENYVLPQYGYLTRGREFDKGTDIVGIADHRSELYFLEHHDAEC 200
PPV3-MK092410 .................................................................................................... 200
PPV3-KU167028 .................................................................................................... 200
PPV3-KU167029 .................................................................................................... 200
PPV3-KY586145 .................................................................................................... 200
PPV3-MK092409 ..........................................................................G......................... 200
PPV3-MK092411 ........................................S........................................................... 200
PPV3-151      .................................R.................................................................. 200
PPV3-155      .................................................................................................... 200
PPV3-829      .................................................................................................... 200
PPV3-838      .................................................................................................... 200

PPV3-MK092412 LGSGDTWSHAYEFPSDLPFRRLTTPNQSLYARHNPIPPSRLAIMTGVDNSGVPKWKRPAGEDVGKHPLNYVPGPSVMMPTDSQIRNTDFRVPLAIGNPVT 300
PPV3-MK092410 .................L.................................................................................. 300
PPV3-KU167028 ...................................................A................................................ 300
PPV3-KU167029 ...................................................A................................................ 300
PPV3-KY586145 .................................................................................................... 300
PPV3-MK092409 ...................................S................................................................ 300
PPV3-MK092411 .................................................................................................... 300
PPV3-151      .................................................................................................... 300
PPV3-155      ...................................................A................................................ 300
PPV3-829      ...................................................A................................................ 300
PPV3-838      ...................................................A................................................ 300

PPV3-MK092412 GDRYSVGPLVHQPWSIRTEEGKSPPDNFAVHSYLGGVAYTRRRHEESYTGHTEEMDGSVTNPSRVVVNEVDMAAPHVGHTFMVPGHTRVDGSGSGSDTVY 400
PPV3-MK092410 ..................G................................................................................. 400
PPV3-KU167028 .........................N.......................................................................... 400
PPV3-KU167029 .........................N.......................................................................... 400
PPV3-KY586145 .........................N.......................................................................... 400
PPV3-MK092409 .........................N..........................K..........................A.....R.............. 400
PPV3-MK092411 ..H......................N.......................................................................... 400
PPV3-151      .........................N.......................................................................... 400
PPV3-155      .........................N.......................................................................... 400
PPV3-829      .........................N.......................................................................... 400
PPV3-838      .........................N.......................................................................... 400

PPV3-MK092412 DPKLYQEPIFPLFPAAVWNPNPLTYDCQIWTKIPDTECRSFAQYPLLGGWGMDAPPPMVFLKMRSQPGPPPGGAHTVPNSNLNQYAIFHLHYTIEREVKR 500
PPV3-MK092410 .......................................F.......................................................W.... 500
PPV3-KU167028 .......................................F.......................................................W.... 500
PPV3-KU167029 .......................................F.......................................................W.... 500
PPV3-KY586145 .......................................F.......................................................W.... 500
PPV3-MK092409 .................R.....................F.......................................................W.... 500
PPV3-MK092411 .............................R.....I...F.......................................................W.... 500
PPV3-151      .......................................F.......................................................W.... 500
PPV3-155      .......................................F.......................................................W.... 500
PPV3-829      .......................................F.......................................................W.... 500
PPV3-838      .......................................F.......................................................W.... 500

PPV3-MK092412 RRRSRRHNPEKPAPFPTTDSGRMPFMLANDDRDPNVPVYEVPSDQWVAQNFSRKL 555
PPV3-MK092410 ....................................................... 555
PPV3-KU167028 ....................................................... 555
PPV3-KU167029 ...................P................................... 555
PPV3-KY586145 ....................................................... 555
PPV3-MK092409 ....................................................... 555
PPV3-MK092411 .................A..................S.................. 555
PPV3-151      ....................................................... 555
PPV3-155      ..............................E........................ 555
PPV3-829      ....................................................... 555
PPV3-838      ..............................E........................ 555
```

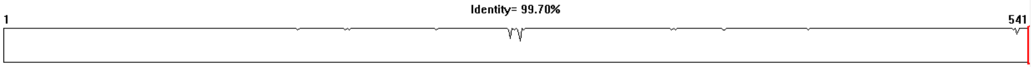


```
PPV4-NC014665 MSGDAIPSIIFNTPWYYYDLNIMSCHFSPSAFQTLIEDYDAFRPRSLTVHLKELVIKDVCQQQGLQAEQVSDNNSATLLAFEDVNYELPYVLGGGQVSVP 100
PPV4-MH921902 .................................................................................................... 100
PPV4-MH921915 .................................................................................................... 100
PPV4-KY586146 .................................................................................................... 100
PPV4-MH921910 .................................................................................................... 100
PPV4-GU978965 .................................................................................................... 100
PPV4-GU978967 .................................................................................................... 100
PPV4-GU978968 .................................................................................................... 100
PPV4-GU978964 .................................................................................................... 100
PPV4-MK092421 .................................................................................................... 100
PPV4-GU978966 .................................................................................................... 100
PPV4-HM031135 .................................................................................................... 100
PPV4-HM031134 .................................................................................................... 100
PPV4-MH921911 .................................................................................................... 100
PPV4-MK092420 .................................................................................................... 100
PPV4-68       .................................................................................................... 100
PPV4-982      .................................................................................................... 100

PPV4-NC014665 GHLPGQPYQLPKYSYRTVGKPDPNSGFVPGRNTHPDQGPGHPKASKTIWYSQYLETQDTEFYILENHKATILHSGNTFSQNYNFPDLPFEQLTQYMWDAR 200
PPV4-MH921902 .................................................................................................... 200
PPV4-MH921915 .................................................................................................... 200
PPV4-KY586146 .................................................................................................... 200
PPV4-MH921910 ................................................................................S................... 200
PPV4-GU978965 .................................................................................................... 200
PPV4-GU978967 .................................................................................................... 200
PPV4-GU978968 .................................................................................................... 200
PPV4-GU978964 .................................................................................................... 200
PPV4-MK092421 .................................................................................................... 200
PPV4-GU978966 .................................................................................................... 200
PPV4-HM031135 .................................................................................................... 200
PPV4-HM031134 .................................................................................................... 200
PPV4-MH921911 .................................................................................................... 200
PPV4-MK092420 .......................................................A..........................T................. 200
PPV4-68       .................................................................................................... 200
PPV4-982      .................................................................................................... 200

PPV4-NC014665 RQDNPLIDQRIQVMSRMYDDGPQKTFAIEVNPYIVPFTVKSTSRPAMFLAGGRFKDGDYSITGPGDREKTSFRYYNDPPWIITRDTYLFSSDLAKTEREQ 300
PPV4-MH921902 ............................K....................................................................... 300
PPV4-MH921915 ............................K....................................................................... 300
PPV4-KY586146 ............................K....................................................................... 300
PPV4-MH921910 ............................K......................................Q....K.C......................... 300
PPV4-GU978965 ............................K......................................Q....K........................... 300
PPV4-GU978967 ............................K......................................Q....K........................... 300
PPV4-GU978968 ............................K......................................Q....K........................... 300
PPV4-GU978964 ............................K......................................Q....K........................... 300
PPV4-MK092421 ............................K......................................Q....K........................... 300
PPV4-GU978966 ............................K......................................Q....K........................... 300
PPV4-HM031135 ............................K......................................Q....K........................... 300
PPV4-HM031134 ............................K......................................Q....K........................... 300
PPV4-MH921911 ............................K......................................Q....K........................... 300
PPV4-MK092420 ............................K.....................................KQ.A.............................. 300
PPV4-68       ............................K....................................................................... 300
PPV4-982      ............................K......................................Q...YK........................... 300

PPV4-NC014665 PGPRQGDTVVRTPDGTLIVTTNALAYGYTTEYLKNIPLLSSKYHGVENFRLAVENERGYSMPGHPSHIRETLFRGKLPSGIRESTIKSEDQRKEITFPDY 400
PPV4-MH921902 ...............................................................................E.................... 400
PPV4-MH921915 ......................................................D........................E.................... 400
PPV4-KY586146 ...............................................................................E.................... 400
PPV4-MH921910 ....................................................A..........................E.................... 400
PPV4-GU978965 ...............................................................................E.................... 400
PPV4-GU978967 ...............................................................................E.................... 400
PPV4-GU978968 ...............................................................................E.................... 400
PPV4-GU978964 ...............................................................................E.................... 400
PPV4-MK092421 ...............................................................................E.................... 400
PPV4-GU978966 ...............................................................................E.................... 400
PPV4-HM031135 ...............................................................................E.................... 400
PPV4-HM031134 ...............................................................................E.................... 400
PPV4-MH921911 ...............................................................................E.................... 400
PPV4-MK092420 ...............................................................................EV................... 400
PPV4-68       ...............................................................................E.................... 400
PPV4-982      ...............................................................................E.................... 400
```

```
PPV4-NC014665 MGSVNEKTTANLESQIWSQIPNTDITEKCTTPPLSIWGMKNPPPMVFLRLLAQMGPPRRSACSGSIPSNTYLNQYCQFLLTYEMEWDVIKRTRKTVRWNP 500
PPV4-MH921902 .................................................................................................... 500
PPV4-MH921915 .................................................................................................... 500
PPV4-KY586146 .................................................................................................... 500
PPV4-MH921910 .................................................................................................... 500
PPV4-GU978965 .................................................................................................... 500
PPV4-GU978967 .................................................................................................... 500
PPV4-GU978968 .................................................................................................... 500
PPV4-GU978964 .................................................................................................... 500
PPV4-MK092421 .................................................................................................... 500
PPV4-GU978966 .................................................................................................... 500
PPV4-HM031135 .................................................................................................... 500
PPV4-HM031134 .................................................................................................... 500
PPV4-MH921911 ........................T........................................................................... 500
PPV4-MK092420 .................................................................................................... 500
PPV4-68       .................................................................................................... 500
PPV4-982      .................................................................................................... 500

PPV4-NC014665 IPPPQIPMGPNNLPVYILNKEGQYRMPTEVWTAKQRPRHRR 541
PPV4-MH921902 ......................................... 541
PPV4-MH921915 ......................................... 541
PPV4-KY586146 ................................T........ 541
PPV4-MH921910 ......................................... 541
PPV4-GU978965 ......................................... 541
PPV4-GU978967 ......................................... 541
PPV4-GU978968 ......................................... 541
PPV4-GU978964 ......................................... 541
PPV4-MK092421 ......................................... 541
PPV4-GU978966 ..................................P...... 541
PPV4-HM031135 ..................................P...... 541
PPV4-HM031134 ..................................P...... 541
PPV4-MH921911 ......................................... 541
PPV4-MK092420 ......................................... 541
PPV4-68       ......................................... 541
PPV4-982      ......................................... 541
```

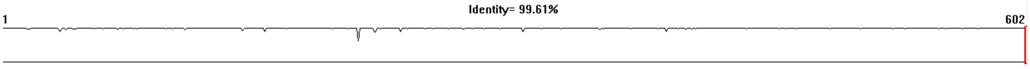


```
PPV5-NC023020 MNEEPPAKRRMTEDRCDSTTRCETLDTQYEDSKMAGGGGGGGNQPKSSWIGGAFFTDTTVTTYGTRRCVLSSFPHNYCTTESGDHIPSLVVCTPWYYYDL 100
PPV5-MK092448 ................................................................................................H... 100
PPV5-MH921905 .................................................................................................... 100
PPV5-MK092441 .................................................................................................... 100
PPV5-MK092434 .................................................................................................... 100
PPV5-MH921912 .................................................................................................... 100
PPV5-MH921908 .................................................................................................... 100
PPV5-MK092452 .................................................................................................... 100
PPV5-JX896319 .................................................................................................... 100
PPV5-JX896318 .................................................................................................... 100
PPV5-MH921904 .................................................................................................... 100
PPV5-MK092451 ................N.....................................L............................................. 100
PPV5-KX273436 ..................................G................................................................. 100
PPV5-KX352458 ..................................G................................................................. 100
PPV5-KX352457 ..................................G................................................................. 100
PPV5-KX352456 ..................................G................................................................. 100
PPV5-KX352455 ..................................G................................................................. 100
PPV5-JX896322 .................................................................................................... 100
PPV5-KU745628 ..............K..................................................................................... 100
PPV5-MK092453 ...............Y.................................................................................... 100
PPV5-MK092449 .....................................V.............................................................. 100
PPV5-MK092438 .....................................S.............................................................. 100
PPV5-MK092435 .............................................................................Y...................... 100
PPV5-MK092426 ...........................................................A........................................ 100
PPV5-MK092425 .................................................................................................... 100
PPV5-MK092430 .................................................................................................... 100
PPV5-MK092450 ...................................................................R................................ 100
PPV5-MK092445 .......................................................................P............................ 100
PPV5-MK092447 ...............................................................................A.................... 100
PPV5-MK092446 ....................................................................A............................... 100
PPV5-JX896321 .................................................................................................... 100
PPV5-JX896320 .................................................................................................... 100
PPV5-MK092439 .................................................................................................... 100
PPV5-MK092431 .................................................................................................... 100
PPV5-MK092436 .................................................................................................... 100
PPV5-MK092427 .................................................................................................... 100
PPV5-MK092443 .................................................................................................... 100
PPV5-MK092444 .................................V.................................................................. 100
PPV5-MK092429 .................................................................................................... 100
PPV5-MK092428 .................................................................................................... 100
PPV5-MK092433 .................................................................................................... 100
PPV5-MK092440 .................................................................................................... 100
PPV5-MK092442 .................................................................................................... 100
PPV5-MK092432 .................................................................................................... 100
PPV5-MK092437 .........................................RES........................................................ 100
PPV5-67       .................................................................................................... 100
PPV5-911      .................................................................................................... 100

PPV5-NC023020 NILSAHFSPSAWQTLLEEYDAFKPLKLEVKIKEIVVKDVNNVTGKQCCDTVSDNAMAAVLCFEDTHYELPYVLGGGQLTVPGHLPGQTYELPKYCYRTVG 200
PPV5-MK092448 Y........................................M.......................................................... 200
PPV5-MH921905 .........................................M.......................................................... 200
PPV5-MK092441 .........................................M.......................................................... 200
PPV5-MK092434 .........................................M.......................................................... 200
PPV5-MH921912 .........................................M.......................................................... 200
PPV5-MH921908 .........................................M.......................................................... 200
PPV5-MK092452 .........................................M.......................................................... 200
PPV5-JX896319 .........................................M.......................................................... 200
PPV5-JX896318 .........................................M.......................................................... 200
PPV5-MH921904 .........................................M.......................................................... 200
PPV5-MK092451 .........................................MA...................................................Y..... 200
PPV5-KX273436 .........................................M............V............................................. 200
PPV5-KX352458 .........................................M............V............................................. 200
PPV5-KX352457 .........................................M............V............................................. 200
PPV5-KX352456 .........................................M............V............................................. 200
PPV5-KX352455 .........................................M............V............................................. 200
PPV5-JX896322 .........................................M.......................................................... 200
PPV5-KU745628 .........................................M.......................................................... 200
PPV5-MK092453 .........................................M.......................................................... 200
PPV5-MK092449 .........................................M.......................................................... 200
PPV5-MK092438 .........................................M.......................................................... 200
PPV5-MK092435 .........................................M.......................................................... 200
PPV5-MK092426 .........................................M.......................................................... 200
PPV5-MK092425 .........................................M.......................................................... 200
PPV5-MK092430 .........................................M.......................................................... 200
PPV5-MK092450 .........................................M.......................................................... 200
PPV5-MK092445 .........................................M.......................................................... 200
PPV5-MK092447 .........................................M.......................................................... 200
PPV5-MK092446 .........................................M.......................................................... 200
PPV5-JX896321 .................................................................................................... 200
PPV5-JX896320 .................................................................................................... 200
PPV5-MK092439 .........................................T.........................G................................ 200
```

PPV5-MK092431 ....M.. 200
PPV5-MK092436 ....M.. 200
PPV5-MK092427 ....M.. 200
PPV5-MK092443 ....M.. 200
PPV5-MK092444 .......P.................................M.. 200
PPV5-MK092429 .......P.................................M.. 200
PPV5-MK092428 ....M.. 200
PPV5-MK092433 ....M.. 200
PPV5-MK092440 ....M.. 200
PPV5-MK092442 ....M.. 200
PPV5-MK092432 ....M.. 200
PPV5-MK092437 ....M.. 200
PPV5-67 ....M.. 200
PPV5-911 ....M.. 200

PPV5-NC023020 KPHSEMWSPIDGSKRAHLDMPFVQPTQNTEFFILENRHSTILHTGNEFFQTYDFPDLHFEQLTQYMWDARRLDNPMKGQRIQVMKNKPTENKDQMFGIRA 300
PPV5-MK092448 ...N.. 300
PPV5-MH921905 300
PPV5-MK092441 300
PPV5-MK092434 300
PPV5-MH921912 ....V.. 300
PPV5-MH921908 ....V.. 300
PPV5-MK092452 ....V.. 300
PPV5-JX896319 ....V.. 300
PPV5-JX896318 ....V.. 300
PPV5-MH921904 .........V........N... 300
PPV5-MK092451 .........V..I..................................... 300
PPV5-KX273436 .........V.........V..............K... 300
PPV5-KX352458 .........V.........V..............K... 300
PPV5-KX352457 .........V.........V..............K... 300
PPV5-KX352456 .........V.........V..............K... 300
PPV5-KX352455 .........V.........V..............K... 300
PPV5-JX896322 .........V.........V.. 300
PPV5-KU745628 ....V.. 300
PPV5-MK092453 ....V.. 300
PPV5-MK092449 ....V.. 300
PPV5-MK092438 300
PPV5-MK092435 ....P... 300
PPV5-MK092426 300
PPV5-MK092425 ....G............................. 300
PPV5-MK092430 ....P............................ 300
PPV5-MK092450 300
PPV5-MK092445 300
PPV5-MK092447 300
PPV5-MK092446 300
PPV5-JX896321 300
PPV5-JX896320 300
PPV5-MK092439 ....A... 300
PPV5-MK092431 300
PPV5-MK092436 ....R....................... 300
PPV5-MK092427 300
PPV5-MK092443 300
PPV5-MK092444 ....L......................................G....... 300
PPV5-MK092429 300
PPV5-MK092428 ....T................ 300
PPV5-MK092433 300
PPV5-MK092440 300
PPV5-MK092442 300
PPV5-MK092432 300
PPV5-MK092437 ....I... 300
PPV5-67 ....V.. 300
PPV5-911 300

PPV5-NC023020 SSYLVPWIVNSLNRPAMFLQGGRLKDGDYSIVGPGTREQATYHYFNDTPVVVERDIYKFTTSMLKRETQQPGPRTQETTVKTPDGTIIITTNSLAYGQVP 400
PPV5-MK092448 400
PPV5-MH921905 400
PPV5-MK092441 400
PPV5-MK092434 400
PPV5-MH921912 400
PPV5-MH921908 400
PPV5-MK092452 400
PPV5-JX896319 400
PPV5-JX896318 400
PPV5-MH921904 400
PPV5-MK092451 400
PPV5-KX273436 ......C...A......... 400
PPV5-KX352458 ......C...A......... 400
PPV5-KX352457 ......C...A......... 400
PPV5-KX352456 ......C...A......... 400
PPV5-KX352455 ......C...A......... 400
PPV5-JX896322 400
PPV5-KU745628 400
PPV5-MK092453 ....I.. 400
PPV5-MK092449 400
PPV5-MK092438 400
PPV5-MK092435 ....A.. 400
PPV5-MK092426 400

```
PPV5-MK092425 .................................................................................................... 400
PPV5-MK092430 .................................................................................................... 400
PPV5-MK092450 .................................................................................................... 400
PPV5-MK092445 .................................................................................................... 400
PPV5-MK092447 .................................................................................................... 400
PPV5-MK092446 .....................................................................R.............................. 400
PPV5-JX896321 .................................................................................................... 400
PPV5-JX896320 .................................................................................................... 400
PPV5-MK092439 .................................................................................................... 400
PPV5-MK092431 .................................................................................................... 400
PPV5-MK092436 .................................................................................................... 400
PPV5-MK092427 .................................................................................................... 400
PPV5-MK092443 ................T.....................................................................A.............. 400
PPV5-MK092444 ............................H....................................................................... 400
PPV5-MK092429 .................................................................................................... 400
PPV5-MK092428 .................................................................................................... 400
PPV5-MK092433 .................................................................................................... 400
PPV5-MK092440 .................................................................................................... 400
PPV5-MK092442 .................................................................................................... 400
PPV5-MK092432 .................................................................................................... 400
PPV5-MK092437 .....................................................G...........................................R.. 400
PPV5-67       .................................................................................................... 400
PPV5-911      .................................................................................................... 400

PPV5-NC023020 ENIDNIPSDHKAAFGVTGYRLAVAEQRGYSTPGMPSHIREILLTKTPKLLEKDQQEITFPNFEGSVSEKTSANLESQIWAYIPNTDNKHNCGTPPLSIWG 500
PPV5-MK092448 .................................................................................................... 500
PPV5-MH921905 .................................................................................................... 500
PPV5-MK092441 .................................................................................................... 500
PPV5-MK092434 .................................................................................................... 500
PPV5-MH921912 .................................................................................................... 500
PPV5-MH921908 .................................................................................................... 500
PPV5-MK092452 .................................................................................................... 500
PPV5-JX896319 .................................................................................................... 500
PPV5-JX896318 .................................................................................................... 500
PPV5-MH921904 .................................................................................................... 500
PPV5-MK092451 .......G............................................................................................ 500
PPV5-KX273436 .................................................................................................... 500
PPV5-KX352458 .................................................................................................... 500
PPV5-KX352457 .................................................................................................... 500
PPV5-KX352456 .................................................................................................... 500
PPV5-KX352455 .................................................................................................... 500
PPV5-JX896322 .................................................................................................... 500
PPV5-KU745628 .................................................................................................... 500
PPV5-MK092453 .................................................................................................... 500
PPV5-MK092449 ..T................................................................................................. 500
PPV5-MK092438 .................................................................................................... 500
PPV5-MK092435 .................................................................................................... 500
PPV5-MK092426 .................................................................................................... 500
PPV5-MK092425 ..................................................................G................................. 500
PPV5-MK092430 .................................................................................................... 500
PPV5-MK092450 .................................................................................................... 500
PPV5-MK092445 .................................................................................................... 500
PPV5-MK092447 .................................................................................................... 500
PPV5-MK092446 .................................................................................................... 500
PPV5-JX896321 .................................................................................................... 500
PPV5-JX896320 .................................................................................................... 500
PPV5-MK092439 .....................................V................................................D............. 500
PPV5-MK092431 .D......................................................T........................................... 500
PPV5-MK092436 .....M.............................................................................................. 500
PPV5-MK092427 .................................................................................................... 500
PPV5-MK092443 .................................................................................................... 500
PPV5-MK092444 .................................................................................................... 500
PPV5-MK092429 .................................................................................................... 500
PPV5-MK092428 ............................................................D....................................... 500
PPV5-MK092433 .................................................................................................... 500
PPV5-MK092440 .................................................................................................... 500
PPV5-MK092442 .................................................................................................... 500
PPV5-MK092432 .................................................................................................... 500
PPV5-MK092437 .................................................................................................... 500
PPV5-67       .................................................................................................... 500
PPV5-911      .................................................................................................... 500

PPV5-NC023020 MENPPPMVFLRLLPQLGPPEKSSCSGSKPSKKFLNQYCQFLLEYTVTWAVVRRKKHTPRWNPMPGVTIPTYNNDPVYILDQNGFYKLPETVWTAKQRVRA 600
PPV5-MK092448 .................................................................................................... 600
PPV5-MH921905 .................................................................................................... 600
PPV5-MK092441 .................................................................................................... 600
PPV5-MK092434 .................................................................................................... 600
PPV5-MH921912 .................................................................................................... 600
PPV5-MH921908 .................................................................................................... 600
PPV5-MK092452 .................................................................................................... 600
PPV5-JX896319 .................................................................................................... 600
PPV5-JX896318 .................................................................................................... 600
PPV5-MH921904 .................................................................................................... 600
PPV5-MK092451 .................................................................................................... 600
PPV5-KX273436 .................................................................................................... 600
PPV5-KX352458 .................................................................................................... 600
PPV5-KX352457 .................................................................................................... 600
```

```
PPV5-KX352456 .................................................................................................... 600
PPV5-KX352455 .................................................................................................... 600
PPV5-JX896322 .................................................................................................... 600
PPV5-KU745628 .................................................................................................... 600
PPV5-MK092453 .................................................................................................... 600
PPV5-MK092449 .................................................................................................... 600
PPV5-MK092438 .................................................................................................... 600
PPV5-MK092435 .................................................................................................... 600
PPV5-MK092426 .................................................................................................... 600
PPV5-MK092425 .................................................................................................... 600
PPV5-MK092430 ..............................R..................................................................... 600
PPV5-MK092450 .........................................................................N.......................... 600
PPV5-MK092445 .................................................................................................... 600
PPV5-MK092447 .......................R............................................................................ 600
PPV5-MK092446 .................................................................................................... 600
PPV5-JX896321 .................................................................................................... 600
PPV5-JX896320 .................................................................................................... 600
PPV5-MK092439 ....S............................................................................................... 600
PPV5-MK092431 .................................................................................................... 600
PPV5-MK092436 .................................................................................................... 600
PPV5-MK092427 ................................................................V................................... 600
PPV5-MK092443 .................................................................................................... 600
PPV5-MK092444 ......................................................R............................................. 600
PPV5-MK092429 .................................................................................................... 600
PPV5-MK092428 .................................................................................................... 600
PPV5-MK092433 .................................................................................................... 600
PPV5-MK092440 .................................................................................................... 600
PPV5-MK092442 .................................................................................................... 600
PPV5-MK092432 .................................................................................................... 600
PPV5-MK092437 ..................................................M................................................. 600
PPV5-67       .................................................................................................... 600
PPV5-911      .................................................................................................... 600

PPV5-NC023020 RR 602
PPV5-MK092448 .. 602
PPV5-MH921905 .. 602
PPV5-MK092441 .. 602
PPV5-MK092434 .. 602
PPV5-MH921912 .. 602
PPV5-MH921908 .. 602
PPV5-MK092452 .. 602
PPV5-JX896319 .. 602
PPV5-JX896318 .. 602
PPV5-MH921904 .. 602
PPV5-MK092451 .. 602
PPV5-KX273436 .. 602
PPV5-KX352458 .. 602
PPV5-KX352457 .. 602
PPV5-KX352456 .. 602
PPV5-KX352455 .. 602
PPV5-JX896322 .. 602
PPV5-KU745628 .. 602
PPV5-MK092453 .. 602
PPV5-MK092449 .. 602
PPV5-MK092438 .. 602
PPV5-MK092435 .. 602
PPV5-MK092426 .. 602
PPV5-MK092425 .. 602
PPV5-MK092430 .. 602
PPV5-MK092450 .. 602
PPV5-MK092445 .. 602
PPV5-MK092447 .. 602
PPV5-MK092446 .. 602
PPV5-JX896321 .. 602
PPV5-JX896320 .. 602
PPV5-MK092439 .. 602
PPV5-MK092431 .. 602
PPV5-MK092436 .. 602
PPV5-MK092427 .. 602
PPV5-MK092443 .. 602
PPV5-MK092444 .. 602
PPV5-MK092429 .. 602
PPV5-MK092428 .. 602
PPV5-MK092433 .. 602
PPV5-MK092440 .. 602
PPV5-MK092442 .. 602
PPV5-MK092432 .. 602
PPV5-MK092437 .. 602
PPV5-67       .. 602
PPV5-911      .. 602
```

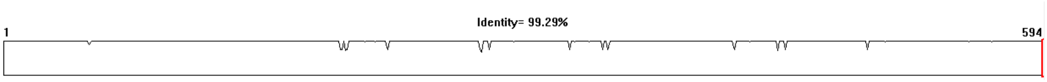


```
PPV6-NC023860 MDISSCCPGGISASGAASNNSGLACGGGGGTNLGTESLVSGCQFGKNSVITSSFRRCLISPWPDKYCCSSAHDLIPGVVYETPWCYYDLNVISAHFSPSA 100
PPV6-MH447535 .................................................................................................... 100
PPV6-MH921907 .................................................................................................... 100
PPV6-MH921900 .................................................................................................... 100
PPV6-MH921901 .................................................................................................... 100
PPV6-MH921903 .................................................................................................... 100
PPV6-KX273435 .................................................................................................... 100
PPV6-KR709262 .................................................................................................... 100
PPV6-KR709263 .................................................................................................... 100
PPV6-MH447539 .................................................................................................... 100
PPV6-MH447540 .................................................................................................... 100
PPV6-MH447537 .................................................................................................... 100
PPV6-KR709267 .................................................................................................... 100
PPV6-MH921909 .................................................V.................................................. 100
PPV6-MH921906 .................................................V.................................................. 100
PPV6-MH447536 .................................................V.................................................. 100
PPV6-MH447538 .................................................V.................................................. 100
PPV6-MH447541 .................................................................................................... 100
PPV6-MH921913 .................................................................................................... 100
PPV6-MH558679 .................................................................................................... 100
PPV6-KR709265 .................................................................................................... 100
PPV6-KR709266 .................................................................................................... 100
PPV6-KR709268 .................................................................................................... 100
PPV6-KF999685 .................................................................................................... 100
PPV6-MK092462 .................................................................................................... 100
PPV6-KR709264 .................................................................................................... 100
PPV6-MG760726 .................................................................................................... 100
PPV6-KY094494 .................................................................................................... 100
PPV6-MK092467 .................................................................................................... 100
PPV6-MK092466 .................................................................................................... 100
PPV6-MK092465 .................................................................................................... 100
PPV6-MK092464 .................................................................................................... 100
PPV6-MK092463 .................................................................................................... 100
PPV6-MK825573 .................................................................................................... 100
PPV6-KF999681 .................................................................................................... 100
PPV6-KF999684 .................................................................................................... 100
PPV6-KF999683 .................................................................................................... 100
PPV6-KF999682 .................................................................................................... 100
PPV6-68       .................................................................................................... 100
PPV6-431      .................................................................................................... 100

PPV6-NC023860 WQRLLEDYDAFRPKSLKVTIQSLVFKDVCQGAEKQTTVQDSQSATIAIFEDKDYDYPYVMGGGQKTVPGHLPGQPYNLPKYSYRTLGSVKESNRASMGGS 200
PPV6-MH447535 .................................................................................................... 200
PPV6-MH921907 .................................................................................................... 200
PPV6-MH921900 .................................................................................................... 200
PPV6-MH921901 .................................................................................................... 200
PPV6-MH921903 .................................................................................................... 200
PPV6-KX273435 .................................................................................................... 200
PPV6-KR709262 .................................................................................................... 200
PPV6-KR709263 .................................................................................................... 200
PPV6-MH447539 .................................................................................................... 200
PPV6-MH447540 .................................................................................................... 200
PPV6-MH447537 .................................................................................................... 200
PPV6-KR709267 .................................................................................................... 200
PPV6-MH921909 .................................................................................................... 200
PPV6-MH921906 .................................................................................................... 200
PPV6-MH447536 .................................................................................................... 200
PPV6-MH447538 .................................................................................................... 200
PPV6-MH447541 .................................................................................................... 200
PPV6-MH921913 .................................................................................................... 200
PPV6-MH558679 .................................................................................................... 200
PPV6-KR709265 .................................................................................................... 200
PPV6-KR709266 .................................................................................................... 200
PPV6-KR709268 .................................................................................................... 200
PPV6-KF999685 .................................................................................................... 200
PPV6-MK092462 .................................................................................................... 200
PPV6-KR709264 .................................................................................................... 200
PPV6-MG760726 .................................................................................................... 200
PPV6-KY094494 ...............................................................................................T.... 200
PPV6-MK092467 ............................................................................................GM.HR... 200
PPV6-MK092466 ............................................................................................GM.HR... 200
PPV6-MK092465 ............................................................................................GM.HR... 200
PPV6-MK092464 ............................................................................................GM.HR... 200
PPV6-MK092463 ............................................................................................GM.HR... 200
PPV6-MK825573 ............................................................................................SM.HH... 200
PPV6-KF999681 ............................................................................................SM.HH... 200
PPV6-KF999684 ............................................................................................SM.HH... 200
PPV6-KF999683 ............................................................................................SM.HH... 200
PPV6-KF999682 ............................................................................................SM.HH... 200
PPV6-68       .................................................................................................... 200
```

```
PPV6-431      .................................................................................................... 200

PPV6-NC023860 GYTFKSNQDTELFLLETHDATLIRGGGTFEQYYEFPNDLPFENLTQYPWDIRRQDNPLYQQRITVMSGSDRDQVGILDGDFYSPFRFKGHDRPAMWLPGQ 300
PPV6-MH447535 .................................................................................................... 300
PPV6-MH921907 .................................................................................................... 300
PPV6-MH921900 .................................................................................................... 300
PPV6-MH921901 .................................................................................................... 300
PPV6-MH921903 .................................................................................................... 300
PPV6-KX273435 .................................................................................................... 300
PPV6-KR709262 .................................................................................................... 300
PPV6-KR709263 .................................................................................................... 300
PPV6-MH447539 .................................................................................................... 300
PPV6-MH447540 .................................................................................................... 300
PPV6-MH447537 .................................................................................................... 300
PPV6-KR709267 .................................................................................................... 300
PPV6-MH921909 ........................................................................T........................... 300
PPV6-MH921906 ........................................................................T........................... 300
PPV6-MH447536 ........................................................................T........................... 300
PPV6-MH447538 ........................................................................T........................... 300
PPV6-MH447541 ........................................................................T........................... 300
PPV6-MH921913 ........................................................................T........................... 300
PPV6-MH558679 ........................................................................T........................... 300
PPV6-KR709265 ........................................................................T........................... 300
PPV6-KR709266 ........................................................................T........................... 300
PPV6-KR709268 ........................................................................T........................... 300
PPV6-KF999685 .................................................................................................... 300
PPV6-MK092462 .................................................................................................... 300
PPV6-KR709264 .........................................................................A.......................... 300
PPV6-MG760726 .........................................................................A.......................... 300
PPV6-KY094494 .........................................................................A.......................... 300
PPV6-MK092467 ............S......S.....................................................A...G...................... 300
PPV6-MK092466 ...................S.....................................................A...G...................... 300
PPV6-MK092465 ...................S.....................................................A...G.............K........ 300
PPV6-MK092464 ...................S.....................................................A...G...................... 300
PPV6-MK092463 ......S............S.....................................................A...G...................... 300
PPV6-MK825573 ...................S.....................................................A...G...................... 300
PPV6-KF999681 ...................S.....................................................A...G...................... 300
PPV6-KF999684 ...................S.....................................................A...G...................... 300
PPV6-KF999683 ...................S.....................................................A...G...................... 300
PPV6-KF999682 ...................S.....................................................A...G...................... 300
PPV6-68       .................................................................................................... 300
PPV6-431      .................................................................................................... 300

PPV6-NC023860 RLIQGKFIDTHPIPNTGRSGVHPNDFHTRGDGHGDTHRTHEERIYSLDTGLAAMPRAAHRPTLQPGPRTLSHAVRRPDGSTVVTANACAYAYTQENPHQE 400
PPV6-MH447535 .................................................................................................... 400
PPV6-MH921907 .................................................................................................... 400
PPV6-MH921900 .................................................................................................... 400
PPV6-MH921901 .................................................................................................... 400
PPV6-MH921903 .................................................................................................... 400
PPV6-KX273435 .................................................................................................... 400
PPV6-KR709262 .................................................................................................... 400
PPV6-KR709263 .................................................................................................... 400
PPV6-MH447539 .................................................................................................... 400
PPV6-MH447540 .................................................................................................... 400
PPV6-MH447537 .................................................................................................... 400
PPV6-KR709267 ..................................E................................................................. 400
PPV6-MH921909 .................................................................................................... 400
PPV6-MH921906 .................................................................................................... 400
PPV6-MH447536 .................................................................................................... 400
PPV6-MH447538 .................................................................................................... 400
PPV6-MH447541 .................................................................................................... 400
PPV6-MH921913 .................................................................................................... 400
PPV6-MH558679 .................................................................................................... 400
PPV6-KR709265 .................................................................................................... 400
PPV6-KR709266 .................................................................................................... 400
PPV6-KR709268 .................................................................................................... 400
PPV6-KF999685 .................................................................................................... 400
PPV6-MK092462 ..........................R......................................................................... 400
PPV6-KR709264 .................................................................................................... 400
PPV6-MG760726 .................................................................................................... 400
PPV6-KY094494 .................................................................................................... 400
PPV6-MK092467 .......................S..................K..T...................................................... 400
PPV6-MK092466 .......................S..................K..T...................................................... 400
PPV6-MK092465 .......................S..................K..T...................................................... 400
PPV6-MK092464 .......................S..................K..T...................................................... 400
PPV6-MK092463 .......................S..................K..T...................................................... 400
PPV6-MK825573 .......................S..................K..T...................................................... 400
PPV6-KF999681 .......................S..................K..T...................................................... 400
PPV6-KF999684 .......................S..................K..T...................................................... 400
PPV6-KF999683 .......................S..................K..T...................................................... 400
PPV6-KF999682 .......................S..................K..T...................................................... 400
PPV6-68       .................................................................................................... 400
PPV6-431      .................................................................................................... 400

PPV6-NC023860 PWSDLNVRHTMYRLAYQRQKGFQQPGDPLHIRTHACYGDGDVTIPKEESLWPTVLGSCTEKSPACLESQIWCKTPNVDMVYGEHTPPLALWGMHAPPPHV 500
PPV6-MH447535 .................................................................................................... 500
PPV6-MH921907 .................................................................................................... 500
```

```
PPV6-MH921900 .................................................................................................... 500
PPV6-MH921901 .................................................................................................... 500
PPV6-MH921903 .................................................................................................... 500
PPV6-KX273435 .................................................................................................... 500
PPV6-KR709262 .................................................................................................... 500
PPV6-KR709263 .................................................................................................... 500
PPV6-MH447539 .................................................................................................... 500
PPV6-MH447540 .................................................................................................... 500
PPV6-MH447537 .................................................................................................... 500
PPV6-KR709267 .................................................................................................... 500
PPV6-MH921909 .................................................................................................... 500
PPV6-MH921906 .................................................................................................... 500
PPV6-MH447536 .................................................................................................... 500
PPV6-MH447538 .................................................................................................... 500
PPV6-MH447541 .................................................................................................... 500
PPV6-MH921913 .................................................................................................... 500
PPV6-MH558679 .................................................................................................... 500
PPV6-KR709265 .................................................................................................... 500
PPV6-KR709266 .................................................................................................... 500
PPV6-KR709268 .................................................................................................... 500
PPV6-KF999685 .................................................................................................... 500
PPV6-MK092462 ..........................G...............A......................................................... 500
PPV6-KR709264 .................................................................................................... 500
PPV6-MG760726 .................................................................................................... 500
PPV6-KY094494 .................................................................................................... 500
PPV6-MK092467 .................H........................S..D...............................................R...... 500
PPV6-MK092466 .................H........................S..D...............................................R...... 500
PPV6-MK092465 .................H........................S..D...............................................R...... 500
PPV6-MK092464 .................H........................S..D...............................................R...... 500
PPV6-MK092463 .................H........................S..D...............................................R...... 500
PPV6-MK825573 .................H........................N..D...............................................R...... 500
PPV6-KF999681 .................H........................N..D...............................................R...... 500
PPV6-KF999684 .................H........................N..D...............................................R...... 500
PPV6-KF999683 .................H........................N..D...............................................R...... 500
PPV6-KF999682 .................H........................S..D...............................................R...... 500
PPV6-68       .................................................................................................... 500
PPV6-431      .................................................................................................... 500

PPV6-NC023860 FLRMLAQEGPPNVSTCRPAQSGQTFINQYGQFLLCFTMVWEVKPRPKSIKQWNPRPPISIPVGQSGPAFILDQDGYYRLPEHVWSARERIRSKR 594
PPV6-MH447535 .............................................................................................. 594
PPV6-MH921907 .............................................................................................. 594
PPV6-MH921900 .............................................................................................. 594
PPV6-MH921901 .............................................................................................. 594
PPV6-MH921903 .............................................................................................. 594
PPV6-KX273435 .............................................................................................. 594
PPV6-KR709262 .............................................................................................. 594
PPV6-KR709263 .............................................................................................. 594
PPV6-MH447539 .............................................................................................. 594
PPV6-MH447540 .............................................................................................. 594
PPV6-MH447537 .............................................................................................. 594
PPV6-KR709267 .............................................................................................. 594
PPV6-MH921909 .............................................................................................. 594
PPV6-MH921906 .............................................................................................. 594
PPV6-MH447536 .............................................................................................. 594
PPV6-MH447538 .............................................................................................. 594
PPV6-MH447541 .............................................................................................. 594
PPV6-MH921913 .............................................................................................. 594
PPV6-MH558679 .............................................................................................. 594
PPV6-KR709265 .............................................................................................. 594
PPV6-KR709266 .............................................................................................. 594
PPV6-KR709268 .............................................................................................. 594
PPV6-KF999685 .............................................................................................. 594
PPV6-MK092462 ...T.......................................................................................... 594
PPV6-KR709264 .............................................................................................. 594
PPV6-MG760726 .............................................................................................. 594
PPV6-KY094494 .............................................................................................. 594
PPV6-MK092467 .............................................................................................. 594
PPV6-MK092466 ..................................................R........................................... 594
PPV6-MK092465 .............................................................................................. 594
PPV6-MK092464 ...............................................................P.............................. 594
PPV6-MK092463 .............................................................................................. 594
PPV6-MK825573 .............................................................................................. 594
PPV6-KF999681 .............................................................................................. 594
PPV6-KF999684 .............................................................................................. 594
PPV6-KF999683 .............................................................................................. 594
PPV6-KF999682 .............................................................................................. 594
PPV6-68       .............................................................................................. 594
PPV6-431      .............................................................................................. 594
```

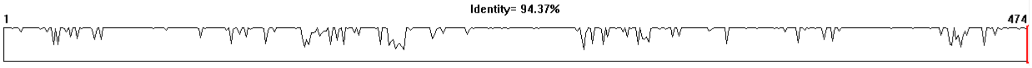


```
PPV7-NC040562 MAEHITLSNTFMAYWENDPYQYPSYVPFQKNNVLSYNTGWHILPNILWRHFLSPKQWYELCINYEAYHVEGTSTTVFNPIPITNNLAIQGTSTFTAFNNT 100
PPV7-MH422967 ....T..................T.K.....E..T.......I......................................................... 100
PPV7-MK092493 .......................T.K.....E..T.......I......................................................... 100
PPV7-MK092482 .......................T.K.....E..T.......I......................................................... 100
PPV7-MK092492 .................A.....T.K.....E..T.......I..........................K.............................. 100
PPV7-MK092488 .......................T.K.....E..T.......I...............................................NT........ 100
PPV7-MT747168 .......................T.K.....E..T................................................................. 100
PPV7-MK092489 .......................T.K.....E..T.......I......................................................... 100
PPV7-MK092484 .......................T.K.....E..T.......I..................................................L...... 100
PPV7-MK092487 .......................T.K.....E..T.......I......................................................... 100
PPV7-MH422966 ....................T..P.....R............I......................................................... 100
PPV7-MH422964 .................A..N..P..........T.......I......................................................... 100
PPV7-MH422965 .................A..N..P..........T.......I......................................................... 100
PPV7-MH422963 .......................T.K.....E..T.......I......................................................... 100
PPV7-MK092496 .......................T.K.....E..T.......I......................................................... 100
PPV7-MG543470 .......................T.K.....K..T.......I......................................................... 100
PPV7-MK484102 .......................A.T...............V.......................................................... 100
PPV7-MK484100 .........................T.....E....S......................................................N........ 100
PPV7-MK092486 .......................A.T.......................................................................... 100
PPV7-MK092483 ........S..............A.T................I......................................................... 100
PPV7-MK092491 .........................T...................V...................................................... 100
PPV7-MG543466 .......................A.T................I......................................................... 100
PPV7-MG543457 .........................T...................V.............................................N........ 100
PPV7-MG543456 .........................T...................V.............................................N........ 100
PPV7-MG543458 .........................T...................V.............................................N........ 100
PPV7-MG543464 .........................T...................V.............................................N........ 100
PPV7-MG543463 .........................T...................V.............................................N........ 100
PPV7-MK092494 ........S................T...................V...................................................... 100
PPV7-MK092479 .........................T...................V.............................I........................ 100
PPV7-MK092478 .........................T...................V.............................I........................ 100
PPV7-MG543462 .........................T...................V.............................................N........ 100
PPV7-MH422962 ........S................T..R................V.............................................N........ 100
PPV7-MK092481 ........................HT...................V.............................I........................ 100
PPV7-MG543469 .........................T...................V.............................................N........ 100
PPV7-MG543468 .........................T...................V.............................................N........ 100
PPV7-MG543459 .........................T................I................................................N........ 100
PPV7-MG902949 .........................K...R.E.............V...................................................... 100
PPV7-MK484101 ........S................T...................V.............................................N........ 100
PPV7-MK092490 .......................A.T........P................................................................. 100
PPV7-MG543472 .............................................V...................................................... 100
PPV7-MG543465 .................................................................................................... 100
PPV7-MG543460 .......................P..........T.......I................................................T........ 100
PPV7-MG543461 .................................................................................................... 100
PPV7-MG543471 .................................................................................................... 100
PPV7-MK092480 ....................T..P.....R...................................................................... 100
PPV7-MK092477 ....................T..P.....R...................................................................... 100
PPV7-MK092485 .......NS...........T..P.....R...................................................................... 100
PPV7-MK092495 ........S...........T..P.....R...................................................................... 100
PPV7-MG543467 .......................P..........T.......I......................................................... 100
PPV7-20       .........................T.......................................................................... 100
PPV7-106      .......................A.T...............V.......................................................... 100
PPV7-152      .........................T.................L.V.............................................N........ 100
PPV7-377      .........................T...................V.............................I...............N........ 100
PPV7-717      .........................T...R.E.................................................................... 100

PPV7-NC040562 IYSLGATDDLYETGYHNWYEDTLWRSWYVAYKEGLVPKRNVTTKEGIGNSWDRLTLPRYLWSAPATAPETNWTWGWNTNKGEKATAYPTAGTTWPHTDSA 200
PPV7-MH422967 .....T................................PTTA....V....S.....T......I.........T....-----KP............AG 200
PPV7-MK092493 .....T................................PTTA....V....S.....T......I.........T....-----KP............AG 200
PPV7-MK092482 .....T................................PTTA....V....S.....T......I.........T....-----KP............AG 200
PPV7-MK092492 .....T.............................I..PTTA....V....S.....T......I.........T....-----KP............AG 200
PPV7-MK092488 .....T................................PTTA....V....S.....T......I.........T....-----KP............AG 200
PPV7-MT747168 ......S...............................PTTA....V....S.....T......I.........T....-----KP............AG 200
PPV7-MK092489 .....T................................PTTA....V....S.....T......I.........T....-----KP............AG 200
PPV7-MK092484 .....T................................PTTA....V....S.....T......I.........T....-----KP............AG 200
PPV7-MK092487 .....TI...............................PTTA....V....S.....T......I.........T....-----KP............AG 200
PPV7-MH422966 .....T...I...........PI..Q............--RSNAG.----....L..T......I.........T....-----KP............AG 200
PPV7-MH422964 .....T...................Q............--RSQVS.AS......L..T....T................-----KP............AG 200
PPV7-MH422965 .....................E.................D.P....V....N..........T.I..............T.P.I.T..P..........G 200
PPV7-MH422963 .....T................................TTTA....V....S.....T......I.........T....-----KP............AG 200
PPV7-MK092496 .....T................................PTTA....V....S.....T......I.........T....-----KP............AG 200
PPV7-MG543470 .....T................................PTTA....V....S.....T......I.........T....-----KP.............G 200
PPV7-MK484102 .....T...............E..................IP....V....N.....K......I.........V...G...G.Q..............G 200
PPV7-MK484100 .....T...............E...................P..D.V....N..L..I.R....I.........V...G...G.Q..............G 200
PPV7-MK092486 .....T........H......E...................P....V....N.....K......I.......I.V...S..DG-...............G 200
PPV7-MK092483 .....T..GI...C.......E...................P....V....N.....K......I.........V...G..DG-...............G 200
PPV7-MK092491 ......I.............NE...................P....V....N............I.........V...G..TG.QN.............G 200
PPV7-MG543466 .....T...............E...................P....V....N.....K......I.........V...G...G.Q..............G 200
PPV7-MG543457 .....F......S........E.................D.P....V....N..L..K......I.........V...G..TG.QN.............G 200
PPV7-MG543456 .....F......S........E.................D.P....V....N..L..K......I.........V...G..TG.QN.............G 200
PPV7-MG543458 .....F......S........E.................D.P....V....N..L..K......I.........V...G..TG.QN.............G 200
PPV7-MG543464 .....F......S........E.................D.P....V....N..L..K......I.........V...G..TG.QN.............G 200
```

```
PPV7-MG543463 .....F......S........E.................D.P....V....N..L..K......I.........V...G..TG.QN.............G 200
PPV7-MK092494 .....F......S........E.................D.P....V....N.....K......I.........V...G..TG.QN.............G 200
PPV7-MK092479 .....F......S........E.................D.P....V....N.....K......I.........V...G..TG.QN.............G 200
PPV7-MK092478 .....F......S........E.................D.P....V....N.....K......I.........V...G..TG.QN.............G 200
PPV7-MG543462 .....T...I...........E.................D.P....V....N..L..K......I.........V...G..TG.QN.............G 200
PPV7-MH422962 .....T...I...........E.................D.P.........N..L..K......I.........V...G..TG.QN.............G 200
PPV7-MK092481 .....F......S........E.................D.P....V....N.....K......I.........V...G..TG.QN.............G 200
PPV7-MG543469 .....F......S........E.................D.P....V....N..L..K......I.........V...G..TG.QN.............. 200
PPV7-MG543468 .....T...I...........E.................D.P....V....N..L..K......I.........V...G..TG.QN.............. 200
PPV7-MG543459 .....F......S........E.................D.P....V....N..L..K......I.........V...G..TG.QN.............. 200
PPV7-MG902949 .....................E.................D.P....V....N............I.........V...G..TG.QN............AG 200
PPV7-MK484101 .....T...I...........E.................D.P....V....N..L..K......I.........V..PG..TG.QN............AG 200
PPV7-MK092490 .....................E.................D.P....V....N............I.........V...G..TG.QN............PG 200
PPV7-MG543472 ..............................................V....................................................G 200
PPV7-MG543465 ...................................................................................................G 200
PPV7-MG543460 .....T.................................D..SQ..AS......LF.T....T................P.----P.............G 200
PPV7-MG543461 .................................................................................................... 200
PPV7-MG543471 .................................................................................................... 200
PPV7-MK092480 ...M.T...I...........PI..Q.............---SDA.G---....L..K....T................TV--PGG..A..........D 200
PPV7-MK092477 ...M.T...I...........PI..Q.............---SNA.G---....L..K....T................TV--PGG..A..........D 200
PPV7-MK092485 ...M.T...I...........PI..Q.............---SNA.G---....L..K....T................TV--PGG..A..........D 200
PPV7-MK092495 ...M.T...I...........PI..Q.............---SNA.G---....L..K....T................TT--PGG.............E 200
PPV7-MG543467 .....T.................................D..SQ..AS......LF.T....T................----PGP.............. 200
PPV7-20       .....F......S........E.................D.P....V....N.....K......I.........V...G..DG-...............G 200
PPV7-106      .....T...............E...................P....V....N.....K......I.........V...G...G.Q..............G 200
PPV7-152      .....F......S........E.................D.P....V....N.....K......I.........V...G..TG.QN.............G 200
PPV7-377      .....F......S........E...................A....V..........K......I.........V...G..TGTQN.............G 200
PPV7-717      .....F......S........E.................D.P....V....N.....K......I.........V...S..TG...............AG 200

PPV7-NC040562 TEQRAAPAGCFWDPLTNPDSIQELRPGKNAMSFHWKTHGADEHCWYNLDSLVKLFPYTPESGYSHNIRDKKYKGPPGSRIVNENFQHPSPQTSISTENHK 300
PPV7-MH422967 ...V..........F...............V.....................................E...Q....T..........T......SDQS. 300
PPV7-MK092493 ...V..........F.....................................................E...Q....T..........T......SDQS. 300
PPV7-MK092482 ...V..........F.....................................................E...Q....T..........T......SDQS. 300
PPV7-MK092492 ...V..........F.....................................................E...Q....T..........T......SDQS. 300
PPV7-MK092488 ...V..........F.....................................................E...Q....T..........T......SDQS. 300
PPV7-MT747168 ...V..........F.....................................................E...Q....T..........T......SDQS. 300
PPV7-MK092489 ...V..........F..................................................S..E...Q....T........R.T......SDQSN 300
PPV7-MK092484 ...V..........F.....................................................E...Q....T..........T......SDQS. 300
PPV7-MK092487 ...V..........F.....................................................E........T..........T......SDQS. 300
PPV7-MH422966 ...V..........F..L..................................................E...Q....T..........T......SDQS. 300
PPV7-MH422964 ...V..........F.....................................................E...Q....T..........T......SDQS. 300
PPV7-MH422965 ...V..........F....................................................K...H.......V...............SDQN. 300
PPV7-MH422963 ...V..........F.....................................................EG..Q..........Y..Q...........L. 300
PPV7-MK092496 ...V...............................................................QN........................F....L. 300
PPV7-MG543470 ...V..........F.........................................................Q....T.V.............F....L. 300
PPV7-MK484102 ...V..........F..................................................S.QN........................F....L. 300
PPV7-MK484100 ...V..........F..................................................S.QN........................F....L. 300
PPV7-MK092486 ...V..........F....................................................QN........................F....L. 300
PPV7-MK092483 ...V..........F..................................................S.QN.....................R..F....L. 300
PPV7-MK092491 ...V..........F....................................................Q.........................F....L. 300
PPV7-MG543466 ...V..........F.....................................................N........................F....L. 300
PPV7-MG543457 ...V..........F.........................................................Q....T.V.............F....L. 300
PPV7-MG543456 ...V..........F.........................................................Q....T.V.............F....L. 300
PPV7-MG543458 ...V..........F.........................................................Q....T.V.............F....L. 300
PPV7-MG543464 ...V..........F.........................................................Q....T.V.............F....L. 300
PPV7-MG543463 ...V..........F.........................................................Q....T.V.............F....L. 300
PPV7-MK092494 ...V..........F.........................................................Q....T.V.............F....L. 300
PPV7-MK092479 ...V..........F.........M.R........................................QN........................F....L. 300
PPV7-MK092478 ...V..........F..............................H.....................QN........................F....L. 300
PPV7-MG543462 ...V..........F..............................................................................F....L. 300
PPV7-MH422962 ...V..........F.....................................................E...Q....T..........T......SDQS. 300
PPV7-MK092481 ...V..........F.....................................................E...Q....T..........T......SDQS. 300
PPV7-MG543469 ...............C....................................................E...Q....T..........T......SDQS. 300
PPV7-MG543468 ...............C.....................................................G................Q.........D.P. 300
PPV7-MG543459 ...V..........F.....................................................EG..Q.............Q...........L. 300
PPV7-MG902949 ...V..........F....................................................QN........................F.S..L. 300
PPV7-MK484101 ...V..........F....................................................QN........................F.S..L. 300
PPV7-MK092490 ...V..........F..................R..................................N...Q....T.V.............F....L. 300
PPV7-MG543472 ...V..........F.........................................................Q....T.V.............F....L. 300
PPV7-MG543465 ...V..........F.........................................................Q....T.V.............F....L. 300
PPV7-MG543460 ...V..........F.........................................................Q....T.V.............F....L. 300
PPV7-MG543461 ...V..........F.....................................................EG..Q.............Q...........L. 300
PPV7-MG543471 .................................................................................................... 300
PPV7-MK092480 P..............................................P....................GG......E.....................Q. 300
PPV7-MK092477 P...................................................................EG......E.....................Q. 300
PPV7-MK092485 P...................................................................EG......E.....................Q. 300
PPV7-MK092495 ...............C.....................................................G................Q.........D.R. 300
PPV7-MG543467 ...............C.....................................................G................Q.........D.P. 300
PPV7-20       ...V..........F....................................................QN........................F....L. 300
PPV7-106      ...V..........F..................................................S.QN........................F....L. 300
PPV7-152      ...V..........F.........................................................Q....T.V.............F....L. 300
PPV7-377      ...V..........F.........................................................Q....T.V.............F....L. 300
PPV7-717      ...V..........F....................................................QN........................F....L. 300

PPV7-NC040562 VFIEHDVPNVLNAPIVPIQWFWIELERNLIEDKKIEKPQLGWPGTEWATPKYPPMNNFIKGIPLTDENGTLVKTVTMGCFRNSIHLSCKKRRSRMFAPTW 400
PPV7-MH422967 .........I........................V................................................................. 400
PPV7-MK092493 .........I........................V................................................................. 400
```

```
PPV7-MK092482 .........I........................V.......................T......................................... 400
PPV7-MK092492 .........I..T.....................V...................................................A............. 400
PPV7-MK092488 .........I..T.....................V...................................................A............. 400
PPV7-MT747168 .........I..T.....................V................................................................. 400
PPV7-MK092489 .........I..T.....................V...................................................A............. 400
PPV7-MK092484 .........I..T.....................V...................................................A............. 400
PPV7-MK092487 .........I..T.....................V...................................................A............. 400
PPV7-MH422966 .........I........................V................................................................. 400
PPV7-MH422964 .........I........................V................................................................. 400
PPV7-MH422965 .................................................................................................... 400
PPV7-MH422963 ..............................................................................Y....L................ 400
PPV7-MK092496 ...................................................................................L................ 400
PPV7-MG543470 ..................................V................................H...........I...L................ 400
PPV7-MK484102 ............T................................................V...................................... 400
PPV7-MK484100 ............T................................................V...................................... 400
PPV7-MK092486 ..........................K..................................V...................................... 400
PPV7-MK092483 ............T................................................V..........N........................... 400
PPV7-MK092491 ............T..............................D.................V...................................... 400
PPV7-MG543466 .................................................................................................... 400
PPV7-MG543457 ..................................V................................H...........I...L................ 400
PPV7-MG543456 ..................................V................................H...........I...L................ 400
PPV7-MG543458 ..................................V................................H...........I...L................ 400
PPV7-MG543464 ..................................V................................H...........I...L................ 400
PPV7-MG543463 ..................................V................................H...........I...L................ 400
PPV7-MK092494 ..................................V................................H...........I...L................ 400
PPV7-MK092479 ...................................................................H.......A...I...L................ 400
PPV7-MK092478 ...................................................................H...........I...L................ 400
PPV7-MG543462 ...................................................................H...........I.................... 400
PPV7-MH422962 .........I..T.....................V.....................D........................................... 400
PPV7-MK092481 .........I........................V................................................................. 400
PPV7-MG543469 .........I........................V................................................................. 400
PPV7-MG543468 ...................................................................K................................ 400
PPV7-MG543459 ...................................................................................L................ 400
PPV7-MG902949 ......I...........................V................................H...........I.................... 400
PPV7-MK484101 ......I...........................V................................H...........I.................... 400
PPV7-MK092490 ..................................V................................H...........I...L................ 400
PPV7-MG543472 ..................................V................................H...........I...L................ 400
PPV7-MG543465 ..................................V................................H...........I...L................ 400
PPV7-MG543460 ..................................V................................H...........I...L................ 400
PPV7-MG543461 ...................................................................................L................ 400
PPV7-MG543471 .................................................................................................... 400
PPV7-MK092480 ...Q......................KS.......................................K..I............................. 400
PPV7-MK092477 ...Q......................KS.......................................K..I............................. 400
PPV7-MK092485 ...Q........T......................................................K..I............................. 400
PPV7-MK092495 ..........................K.....................I..................K..I............................. 400
PPV7-MG543467 ...................................................................K................................ 400
PPV7-20       ...................................................................................L................ 400
PPV7-106      ............T................................................V...................................... 400
PPV7-152      ..................................V................................H...........I...L................ 400
PPV7-377      ..................................V................................H...........I...L................ 400
PPV7-717      ..........................K........................................................L................ 400

PPV7-NC040562 GPMSVEMTHGIDSAFVLPTVRYRTGGARRSWQAKTRDAKDGQTEQTWYQWNPYGTGTYTSTTTSTYTTTTSRK 474
PPV7-MH422967 .................................R...TR.KEPQ.P.......M....S............... 474
PPV7-MK092493 .................................R...TR.KEPQ.P.......M....S............... 474
PPV7-MK092482 .................................R...TR.KESQ.P.......M....S............... 474
PPV7-MK092492 .................................R...TR.KEAQ.P.......M....S............... 474
PPV7-MK092488 .................................R...TR.KEPQ.P.......M....S.....N......... 474
PPV7-MT747168 .................................R...TR.KEPQ.P.......M....S............... 474
PPV7-MK092489 .................................R...TR.K.PQ.P.......M.......A............ 474
PPV7-MK092484 .................................R...VR.Q.PQ.P.......M..A................. 474
PPV7-MK092487 .................................R...VR.Q.QQ.P.......E.................... 474
PPV7-MH422966 .................................R...TR.KEPQ.P.......M....S............... 474
PPV7-MH422964 .................................R...TR.KEPQ.P.......M....S............... 474
PPV7-MH422965 .................................R...VR.Q.QQ.P.......M.................... 474
PPV7-MH422963 ........................................Q....P.......K.................... 474
PPV7-MK092496 .................................R...VR.Q.QQ.P.......E.................... 474
PPV7-MG543470 .................................R...T..Q..Q.P............................ 474
PPV7-MK484102 .................................R...VR.QHQHHS............................ 474
PPV7-MK484100 .................................R...VR.QHQHHS............................ 474
PPV7-MK092486 .................................R..GVR.Q.QQ.P.......E......A............. 474
PPV7-MK092483 .................................R...VR.Q.QQ.P.......E.................... 474
PPV7-MK092491 ..........T......................R...VR.K.QQ.P.......E.................... 474
PPV7-MG543466 ........................................Q..Q.P............................ 474
PPV7-MG543457 .................................R...T..Q..Q.P............................ 474
PPV7-MG543456 .................................R...T..Q..Q.P............................ 474
PPV7-MG543458 .................................R...T..Q..Q.P............................ 474
PPV7-MG543464 .................................R...T..Q..Q.P............................ 474
PPV7-MG543463 .................................R...T..Q..Q.P............................ 474
PPV7-MK092494 .................................R...T..Q..Q.P............................ 474
PPV7-MK092479 .................................R...T..Q.P..P............................ 474
PPV7-MK092478 .................................R...T..Q.P..P............................ 474
PPV7-MG543462 .................................R...T..Q....P............................ 474
PPV7-MH422962 .................................R...TR.KEPQ.P.......M....S............... 474
PPV7-MK092481 ............................G....R...TR.KEPQ.P.......M....S............... 474
PPV7-MG543469 .......................................................................... 474
PPV7-MG543468 .......................................................................... 474
```

```
PPV7-MG543459 ........................................Q...P...........................  474
PPV7-MG902949 .................................R...VR.Q.QQ.P.......M.................Q.  474
PPV7-MK484101 .................................R...VR.Q.QQ.P.......M...................  474
PPV7-MK092490 ....................G............R...VR.Q.QQ.P.......E...................  474
PPV7-MG543472 .................................R...T..Q..Q.P...........................  474
PPV7-MG543465 .................................R...T..Q..Q.P...........................  474
PPV7-MG543460 .................................R...T..Q..Q.P...........................  474
PPV7-MG543461 .................................R...T..Q..Q.P...........................  474
PPV7-MG543471 ..........................................................................  474
PPV7-MK092480 .....................................VQ.Q.P.KR......K...S..........I....  474
PPV7-MK092477 .....................................VQ.Q.P.KR......K...S..........I....  474
PPV7-MK092485 .....................................VQ.Q.P.KR......M.......A...........  474
PPV7-MK092495 .............................................S...............A...........  474
PPV7-MG543467 ..........................................................................  474
PPV7-20       .................................R...VR.Q.QQ.P.......E...................  474
PPV7-106      .................................R...VR.Q.PQ.S.......E...................  474
PPV7-152      .................................R...T..Q..Q.P...........................  474
PPV7-377      .................................R...T..Q..Q.P...........................  474
PPV7-717      .................................R...VR.Q.QQ.P.......M...................  474
```
